# Supplementary material for: First crAss-Like Phage Genome Encoding the Diversity-Generating Retroelement (DGR)
Source: Viruses. 2020 May 22;12(5):573. doi: 10.3390/v12050573 (PMC7290462; doi:10.3390/v12050573)
Supplement: Supplementary file 1 [file viruses-12-00573-s001.zip › Fig. S1.pdf]

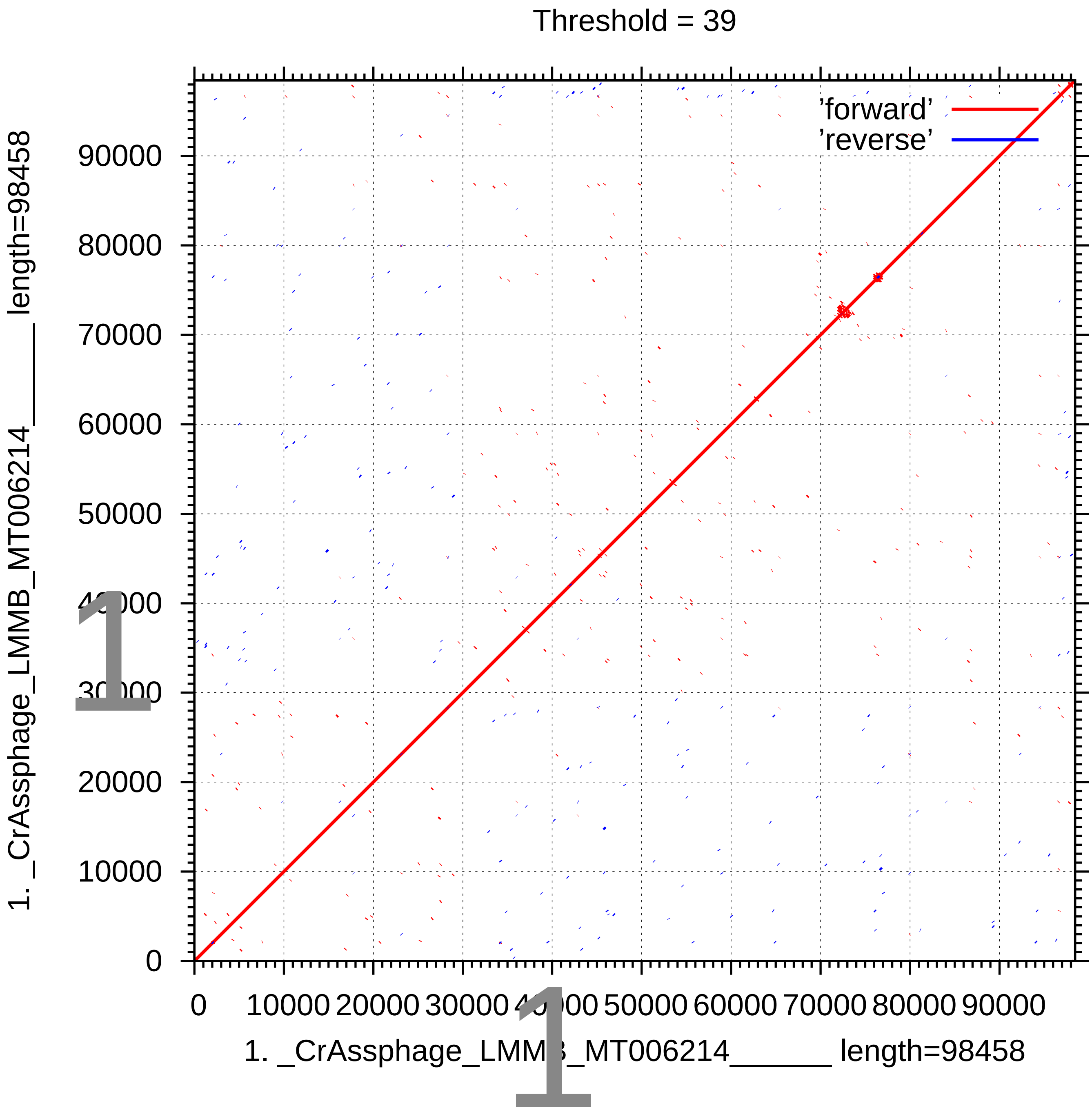
Threshold = 39
'forward'
'reverse'
90000
80000
70000
60000
50000
40000
30000
20000
10000
0
1. _CrAssphage_LMMB_MT006214______ length=98458
0
10000
20000
30000
40000
50000
60000
70000
80000
90000
1. _CrAssphage_LMMB_MT006214______ length=98458
1
1

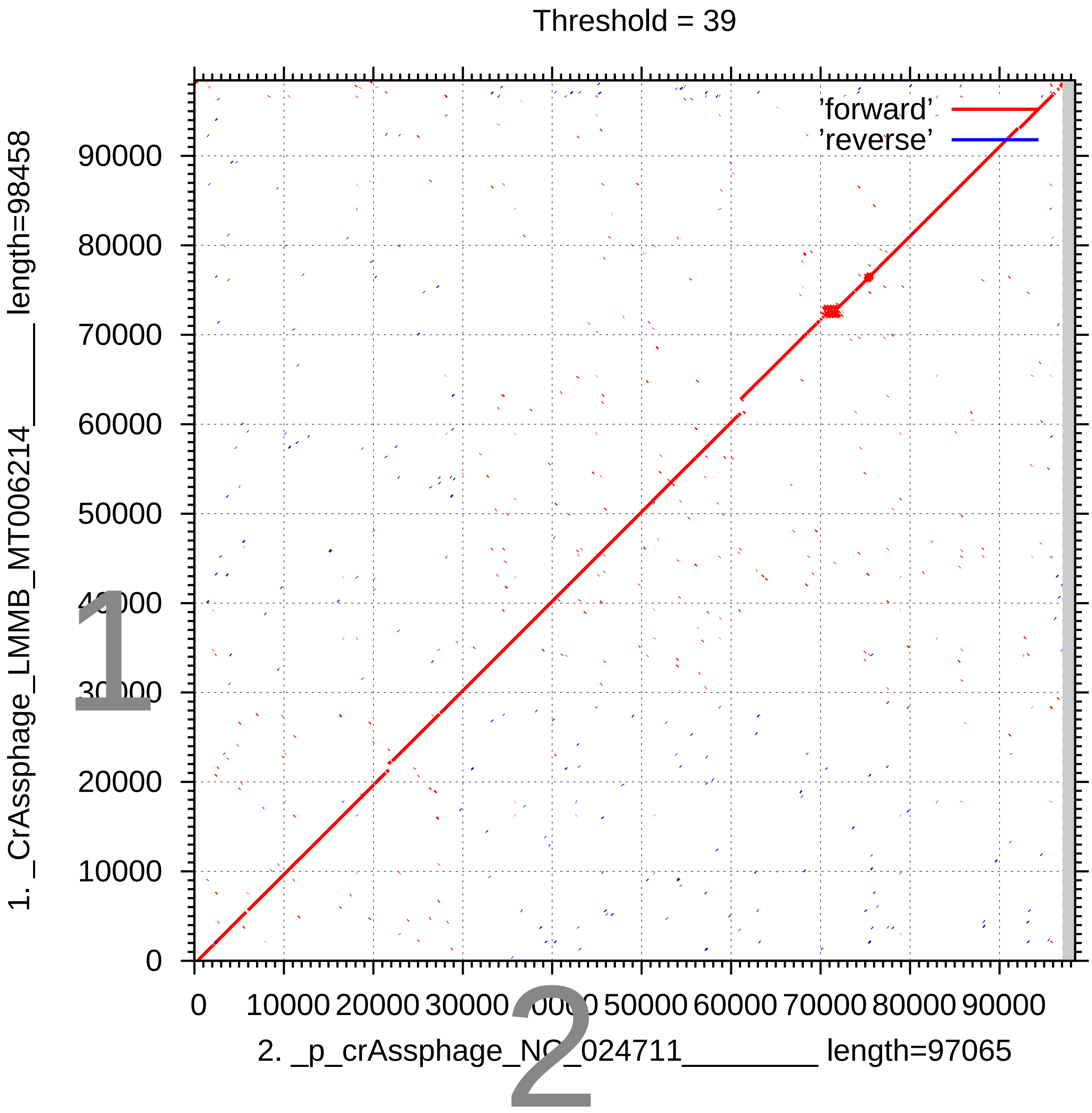
Threshold = 39
'forward'
'reverse'
1. _CrAssphage_LMMB_MT006214________ length=98458
2. _p_crAssphage_NC_024711________ length=97065
0
10000
20000
30000
40000
50000
60000
70000
80000
90000
1
2

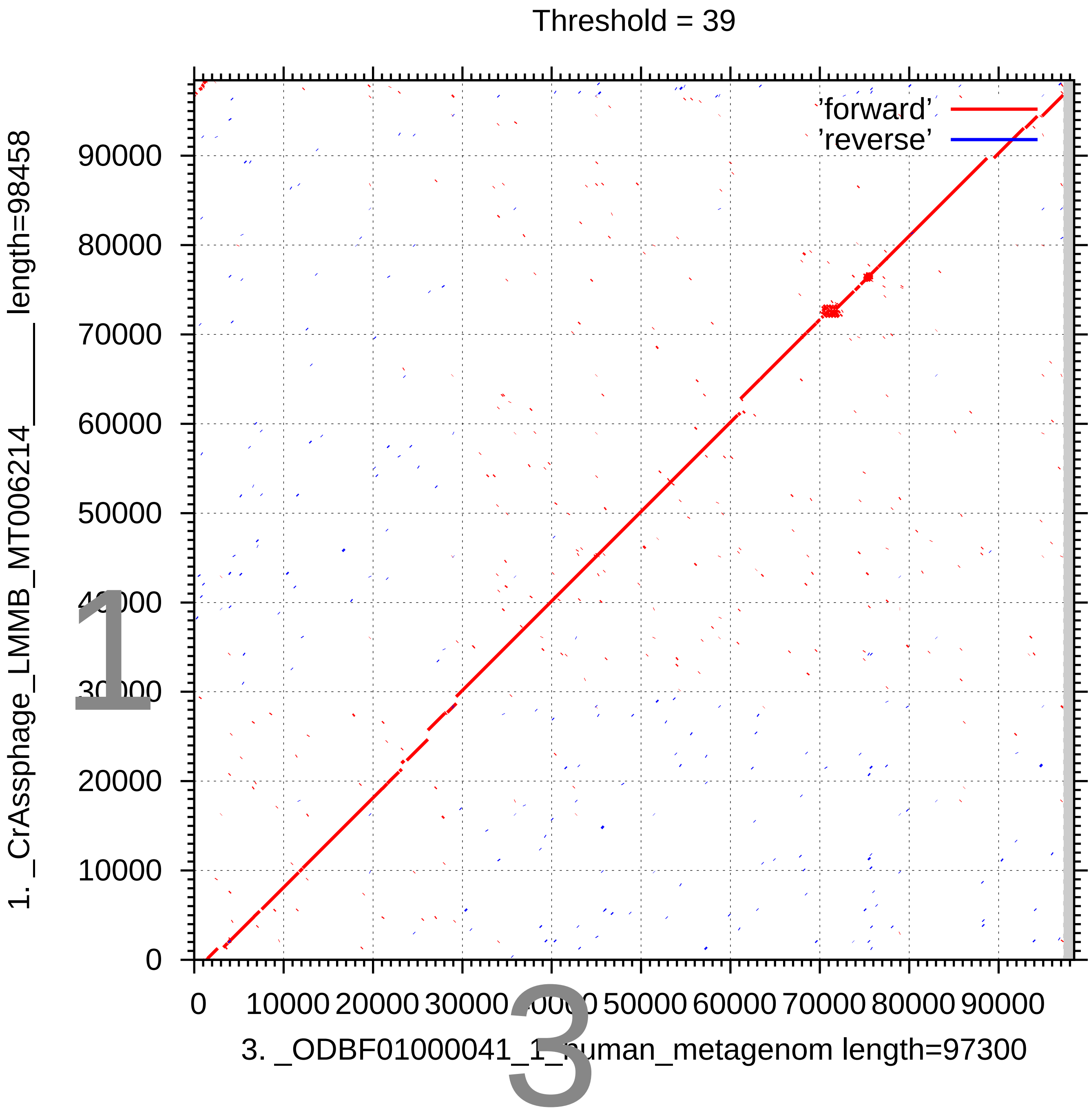

Threshold = 39
'forward'
'reverse'
1. _CrAssphage_LMMB_MT006214 length=98458
3. _ODBF01000041_1_human_metagenom length=97300
0
10000
20000
30000
40000
50000
60000
70000
80000
90000

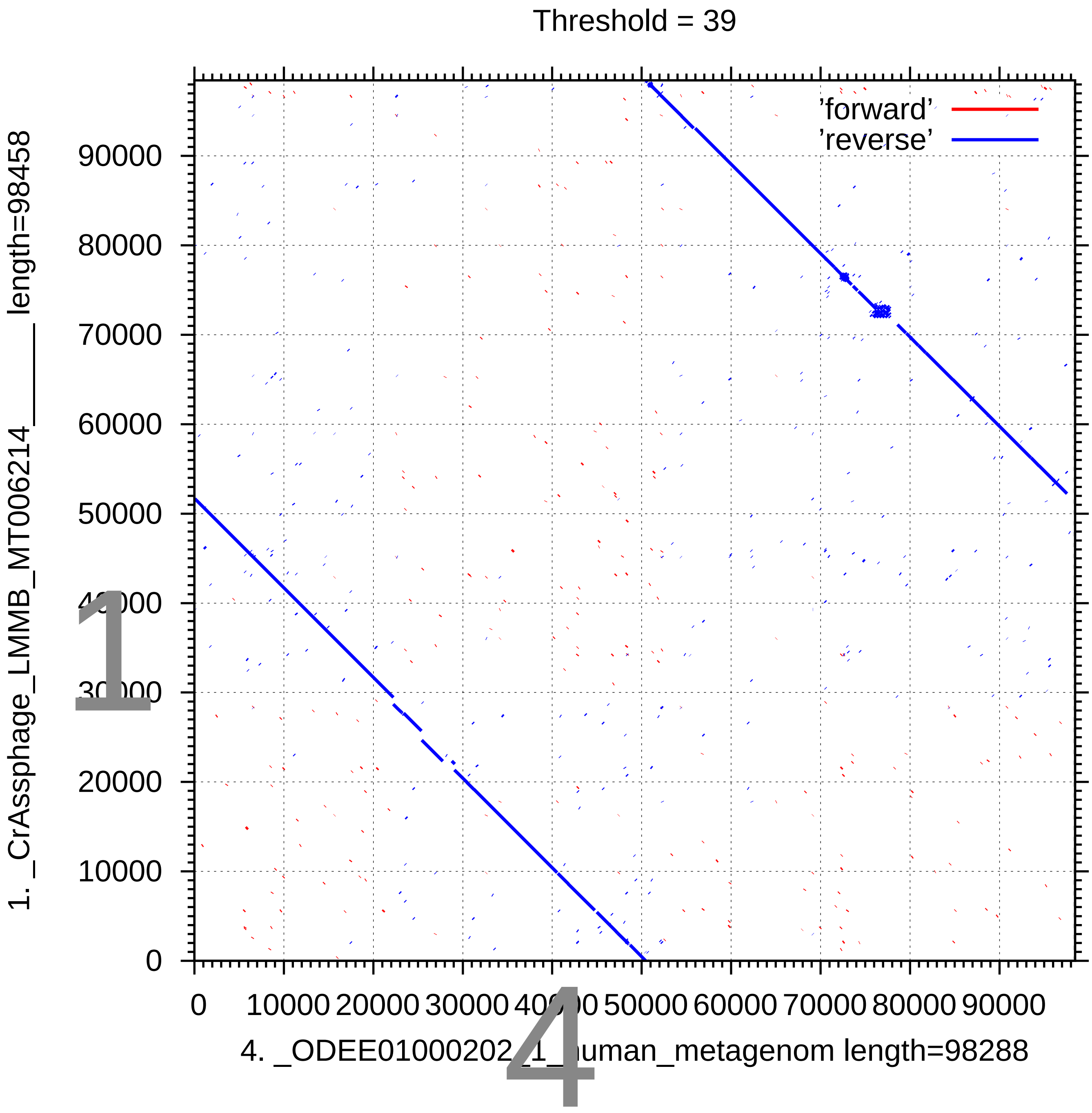
Threshold = 39
'forward'
'reverse'
1. _CrAssphage_LMMB_MT006214________ length=98458
4. _ODEE01000202_1_human_metagenom length=98288
0
10000
20000
30000
40000
50000
60000
70000
80000
90000
1
4

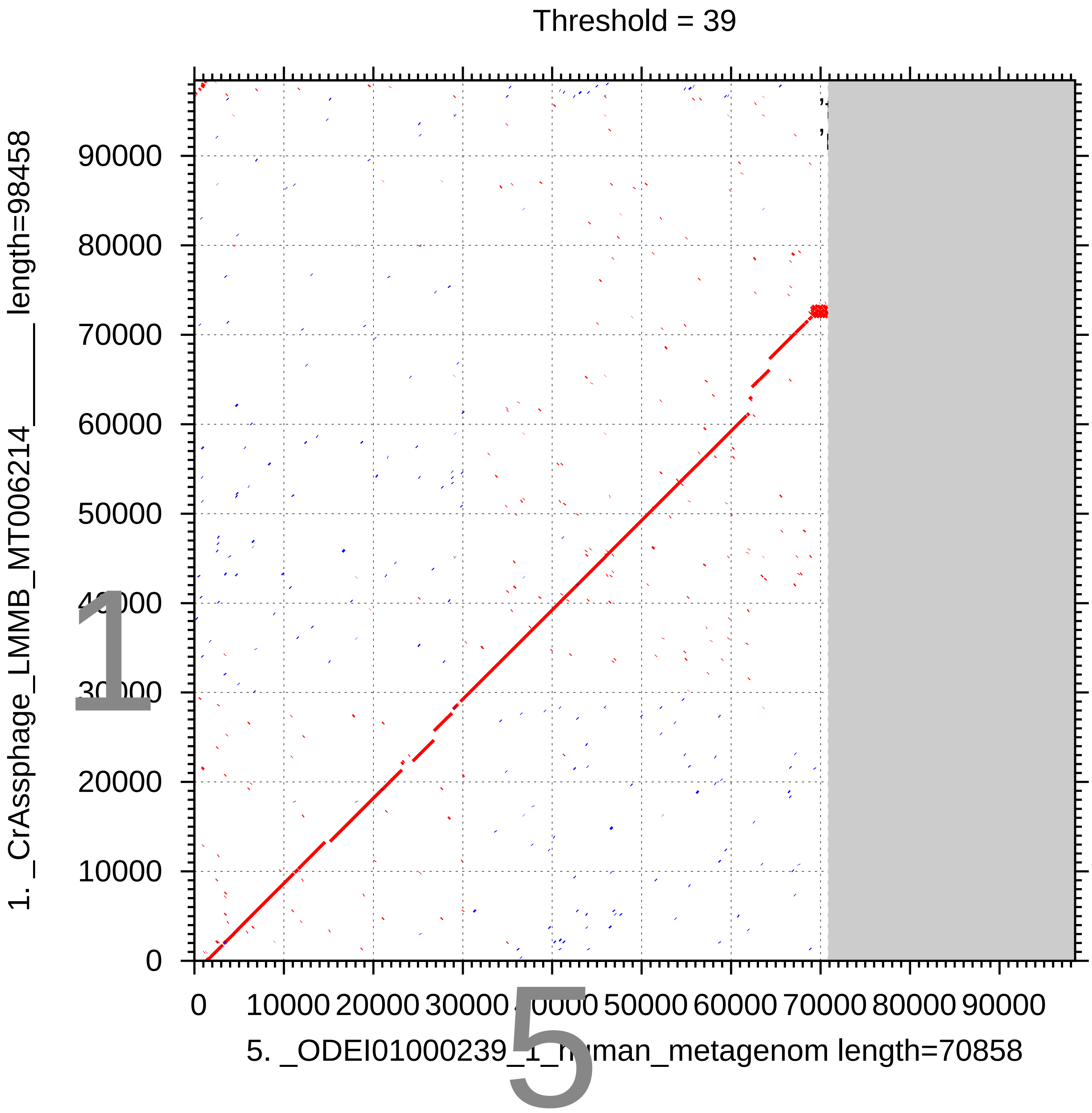

Threshold = 39
1. _CrAssphage_LMMB_MT006214_______ length=98458
5. _ODEI01000239_1_human_metagenom length=70858
0
10000
20000
30000
40000
50000
60000
70000
80000
90000
1
5

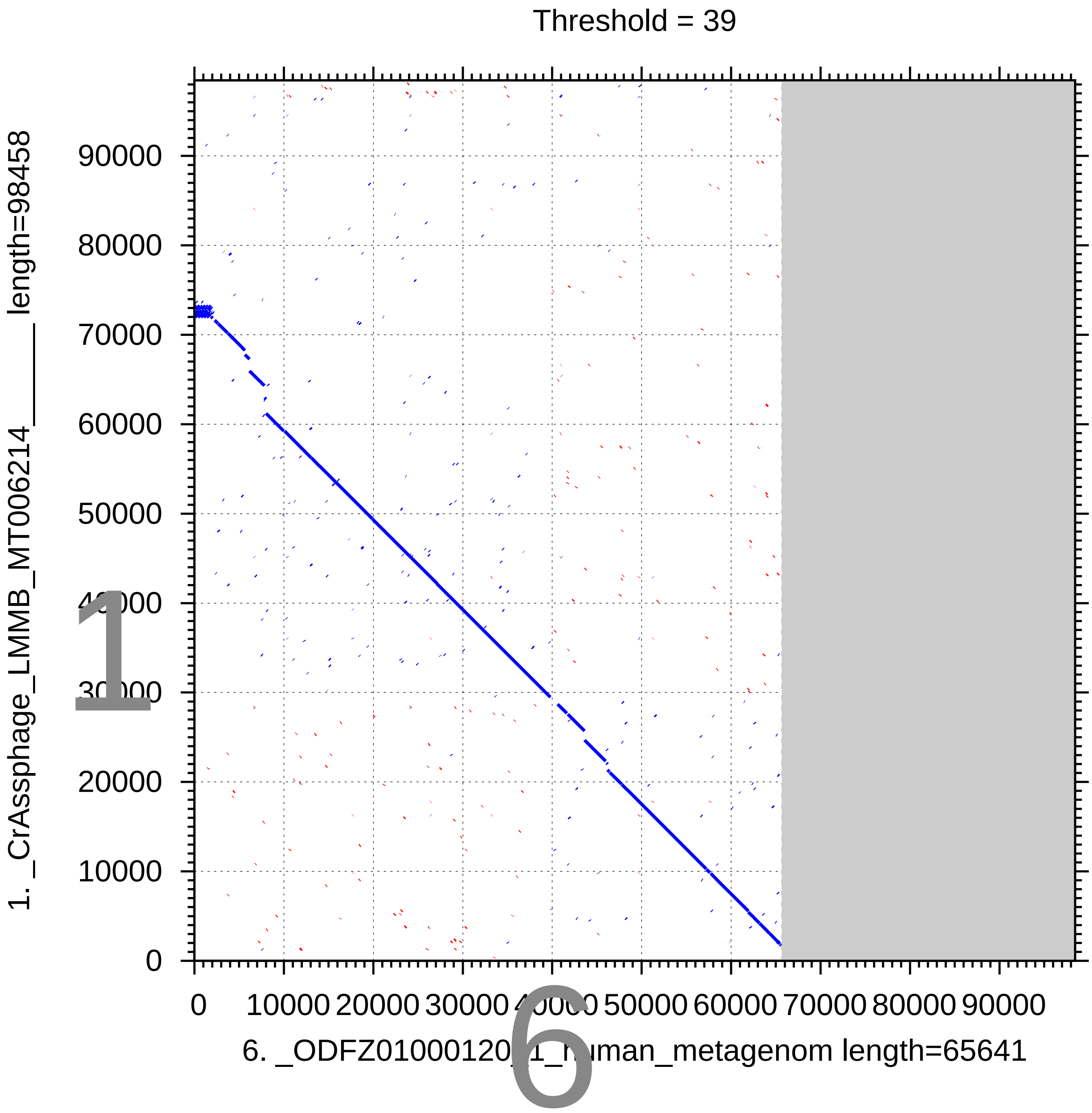

Threshold = 39
1. _CrAssphage_LMMB_MT006214_________ length=98458
6. _ODFZ01000120_1_human_metagenom length=65641
0
10000
20000
30000
40000
50000
60000
70000
80000
90000
1
6

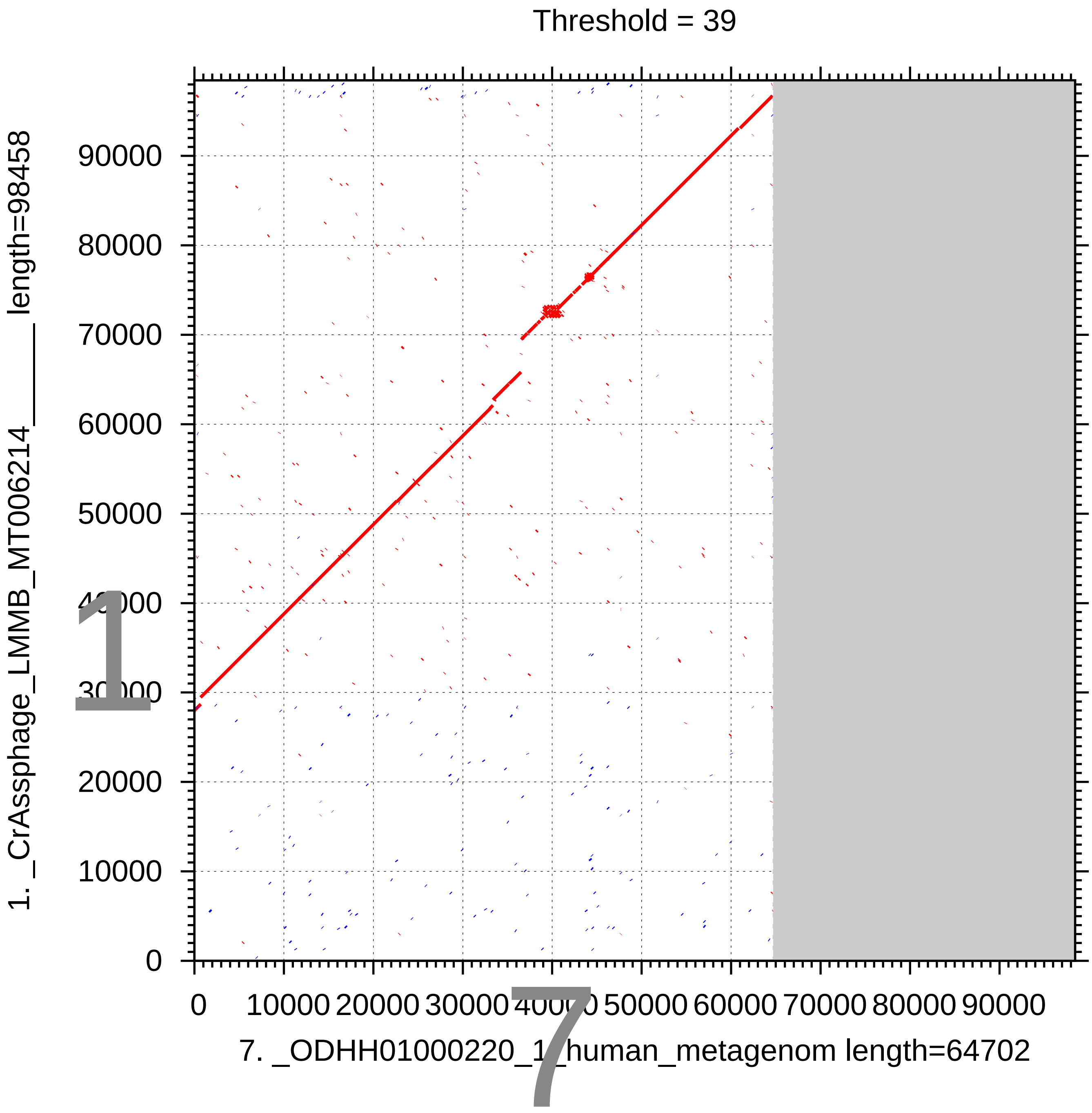
Threshold = 39
1. _CrAssphage_LMMB_MT006214_______ length=98458
7. _ODHH01000220_1_human_metagenom length=64702
0
10000
20000
30000
40000
50000
60000
70000
80000
90000
1
7

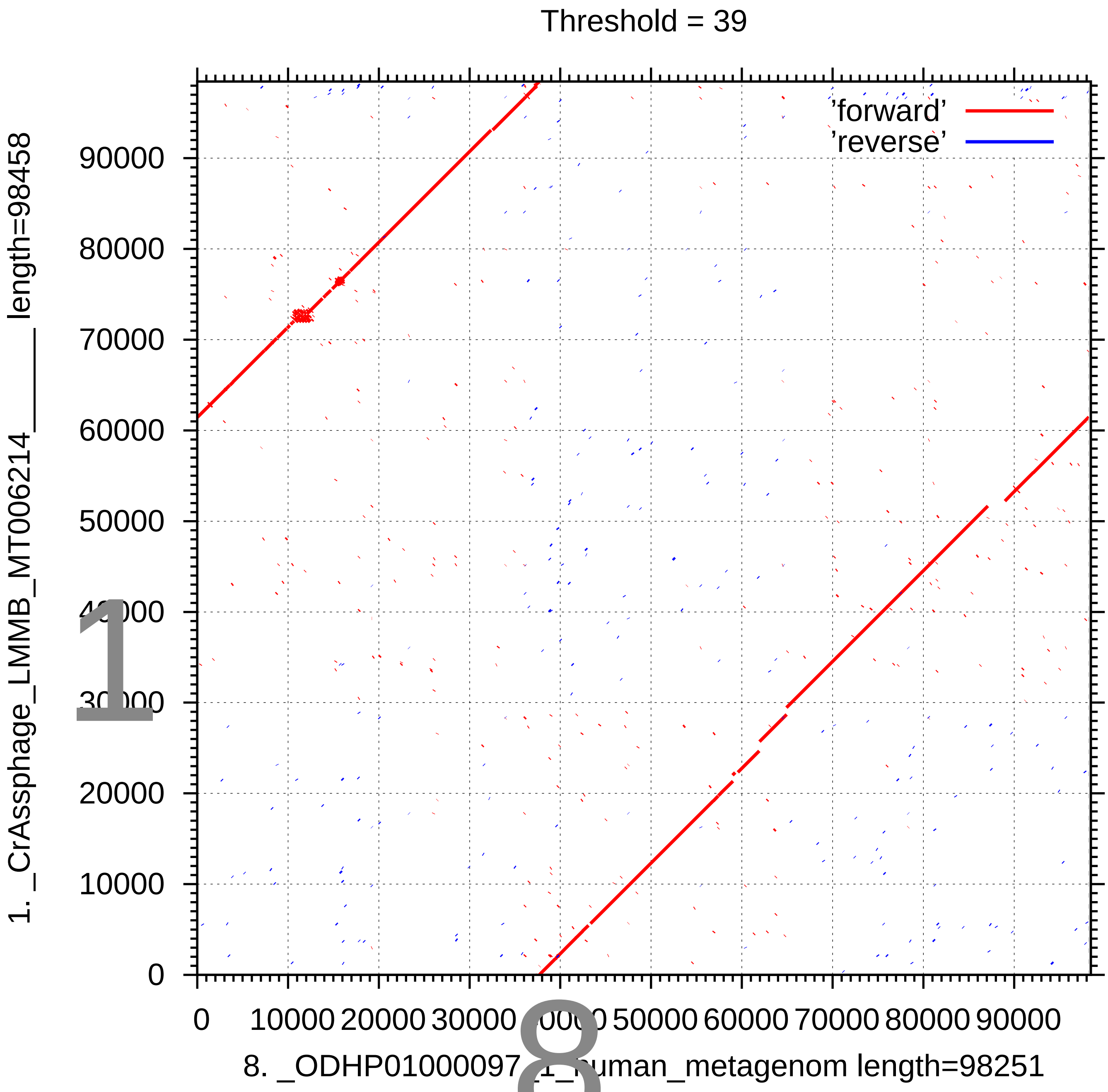

Threshold = 39
'forward'
'reverse'
1. _CrAssphage_LMMB_MT006214_______length=98458
8. _ODHP01000097_1_human_metagenom length=98251
0
10000
20000
30000
50000
60000
70000
80000
90000

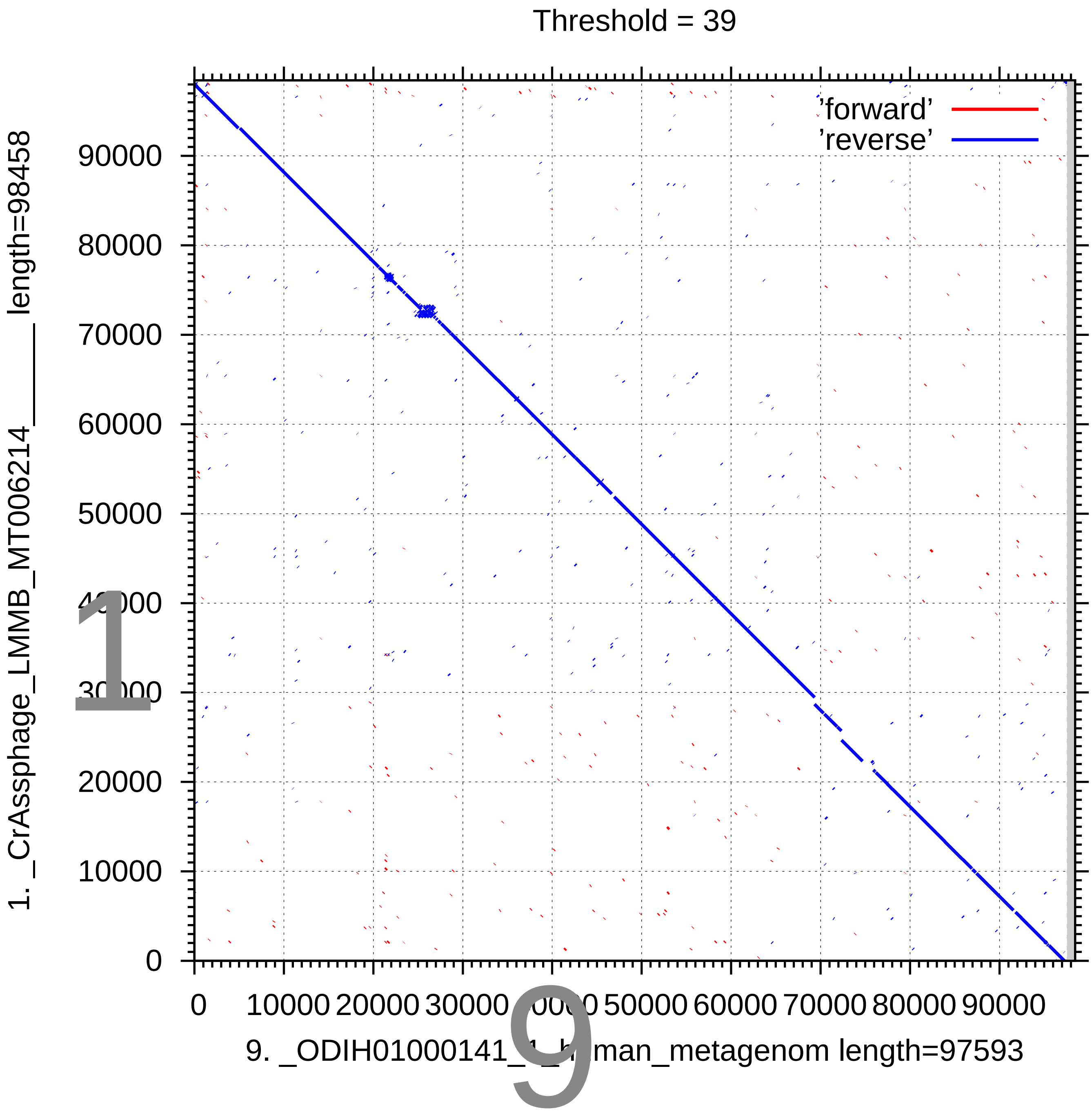
Threshold = 39
'forward'
'reverse'
90000
80000
70000
60000
50000
40000
30000
20000
10000
0
0
10000
20000
30000
40000
50000
60000
70000
80000
90000
1. _CrAssphage_LMMB_MT006214_______ length=98458
9. _ODIH01000141_1_human_metagenom length=97593

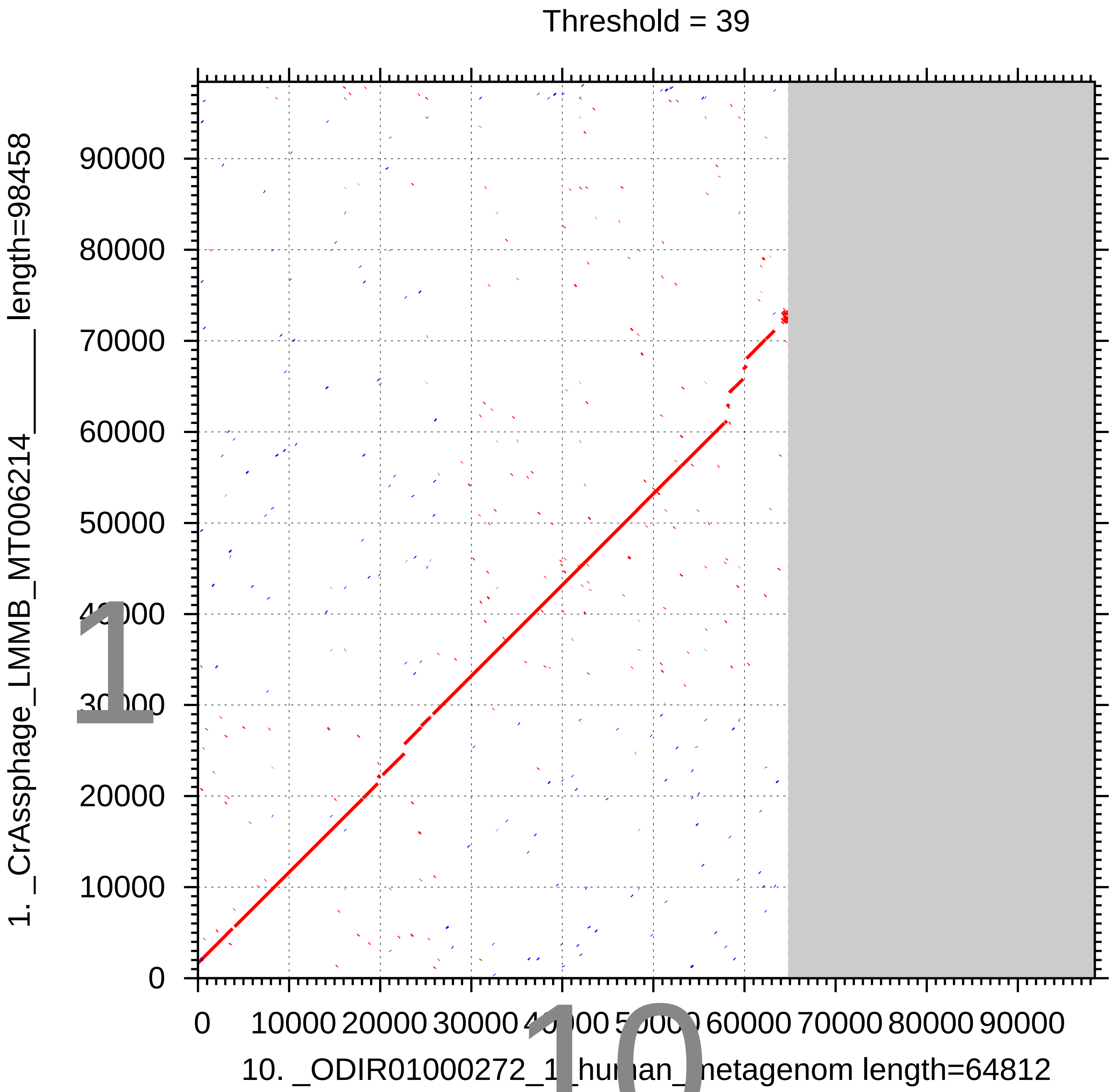

Threshold = 39
1. _CrAssphage_LMMB_MT006214_______length=98458
10. _ODIR01000272_1_human_metagenom length=64812
0
10000
20000
30000
40000
50000
60000
70000
80000
90000

1

10

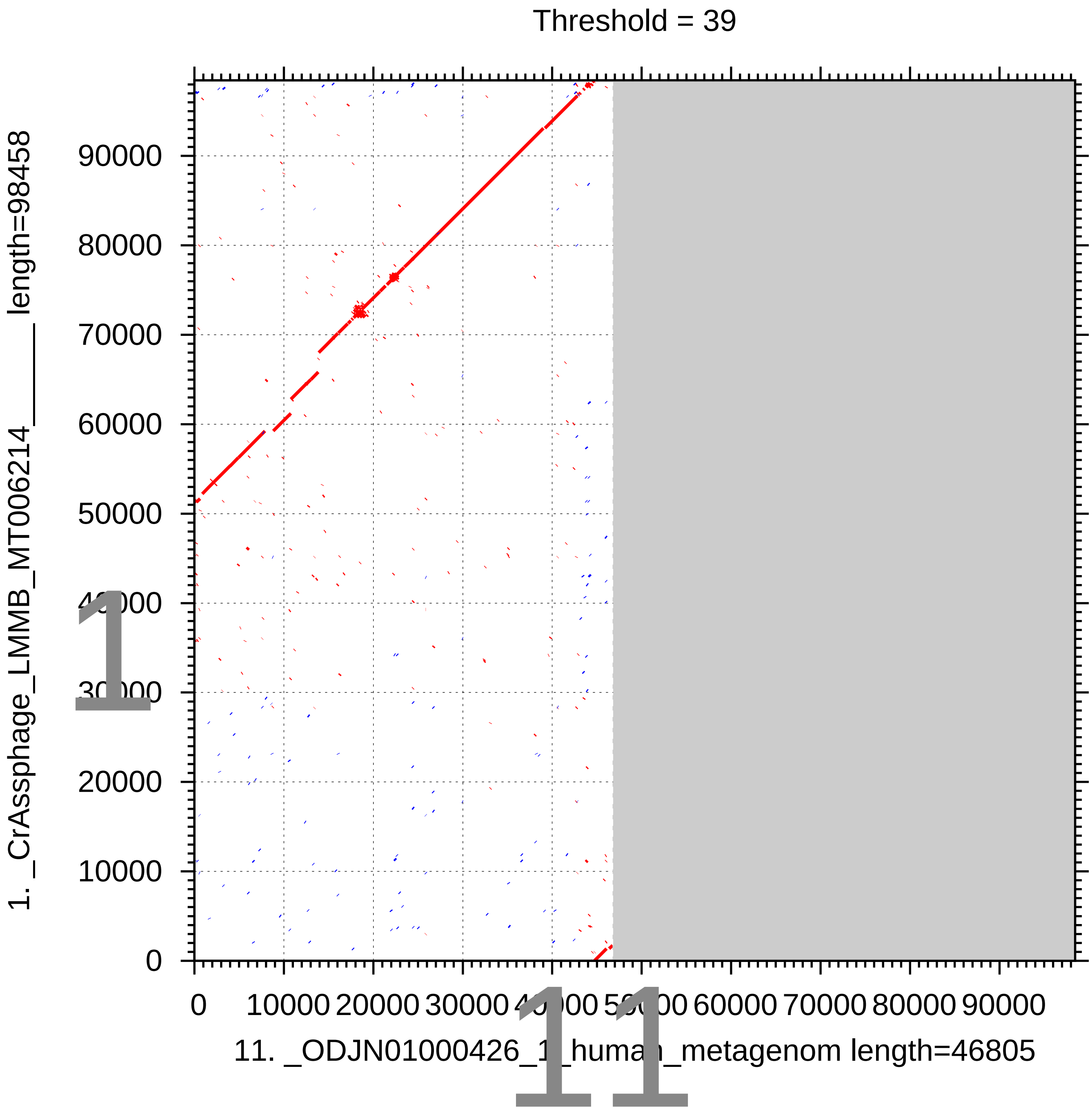
Threshold = 39
1. _CrAssphage_LMMB_MT006214________length=98458
90000
80000
70000
60000
50000
40000
30000
20000
10000
0
0
10000
20000
30000
40000
50000
60000
70000
80000
90000
11. _ODJN01000426_1_human_metagenom length=46805
1
11

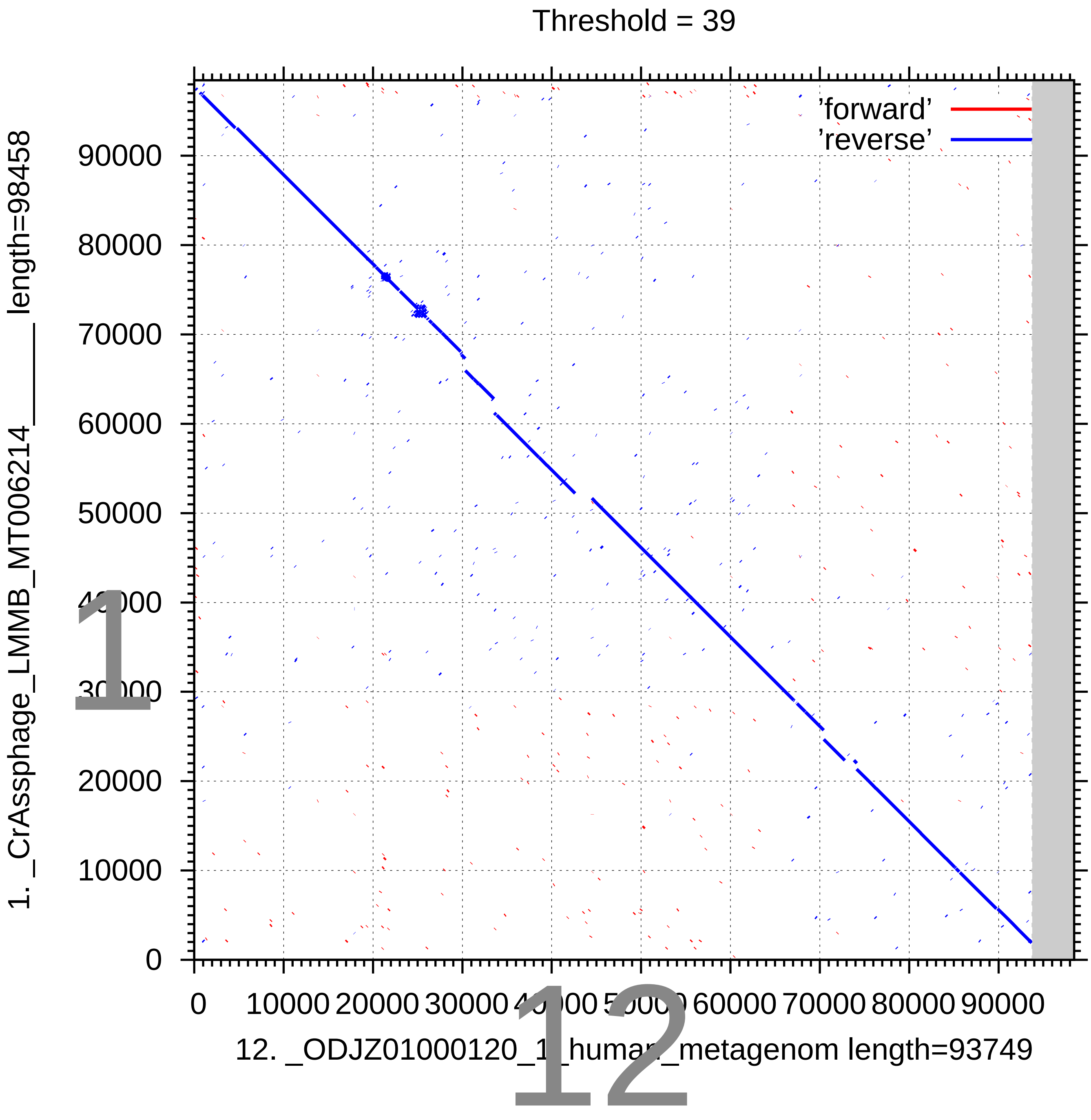

Threshold = 39
'forward'
'reverse'
90000
80000
70000
60000
50000
40000
30000
20000
10000
0
0
10000
20000
30000
40000
50000
60000
70000
80000
90000
12. _ODJZ01000120_1_human_metagenom length=93749
1. _CrAssphage_LMMB_MT006214_______length=98458
1
12

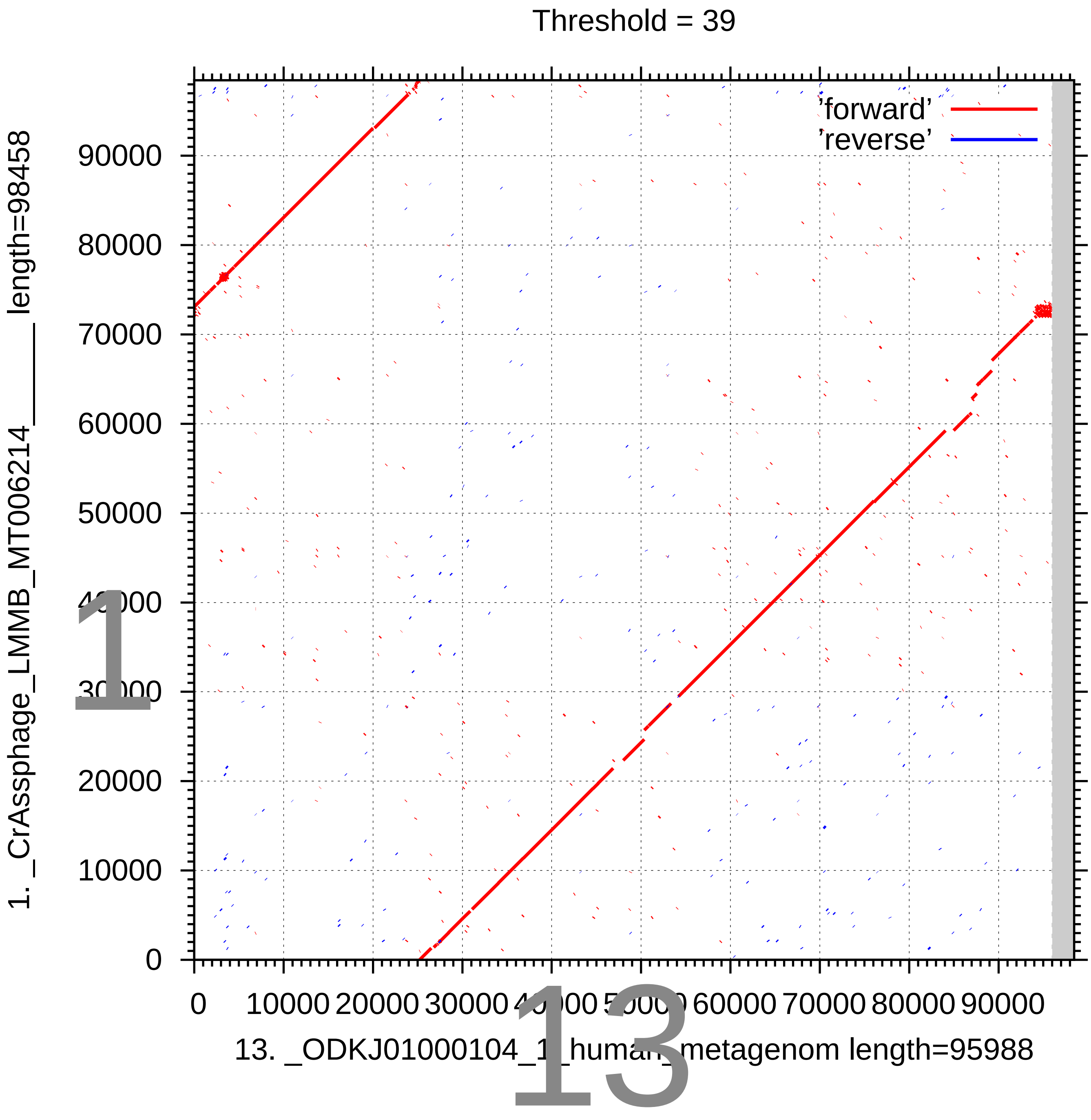

Threshold = 39
'forward'
'reverse'
1. _CrAssphage_LMMB_MT006214_______ length=98458
13. _ODKJ01000104_1_human_metagenom length=95988
0
10000
20000
30000
40000
50000
60000
70000
80000
90000
1
13

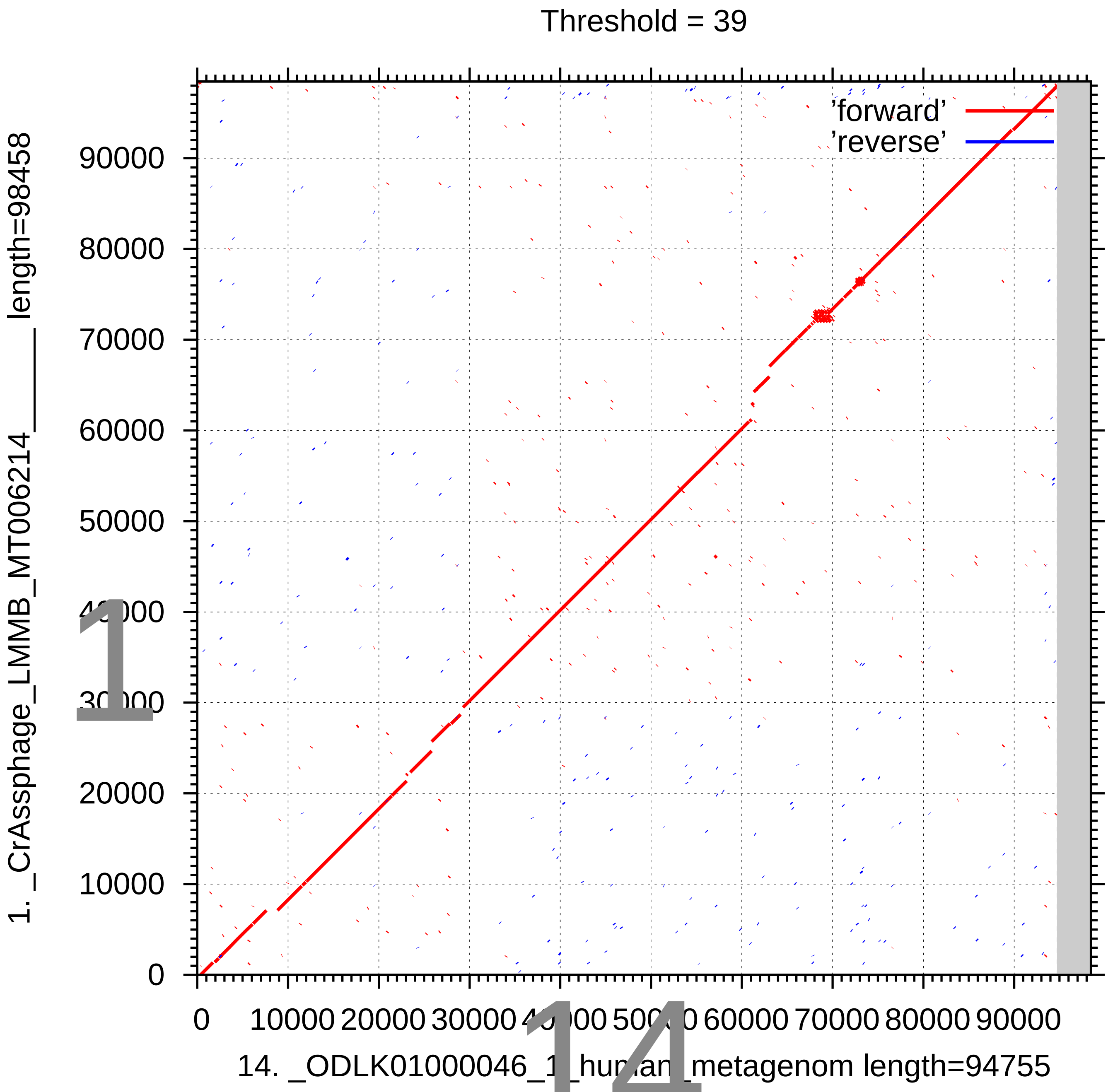

Threshold = 39
'forward'
'reverse'
1. _CrAssphage_LMMB_MT006214_______ length=98458
14. _ODLK01000046_1_human_metagenom length=94755
0
10000
20000
30000
40000
50000
60000
70000
80000
90000

1

14

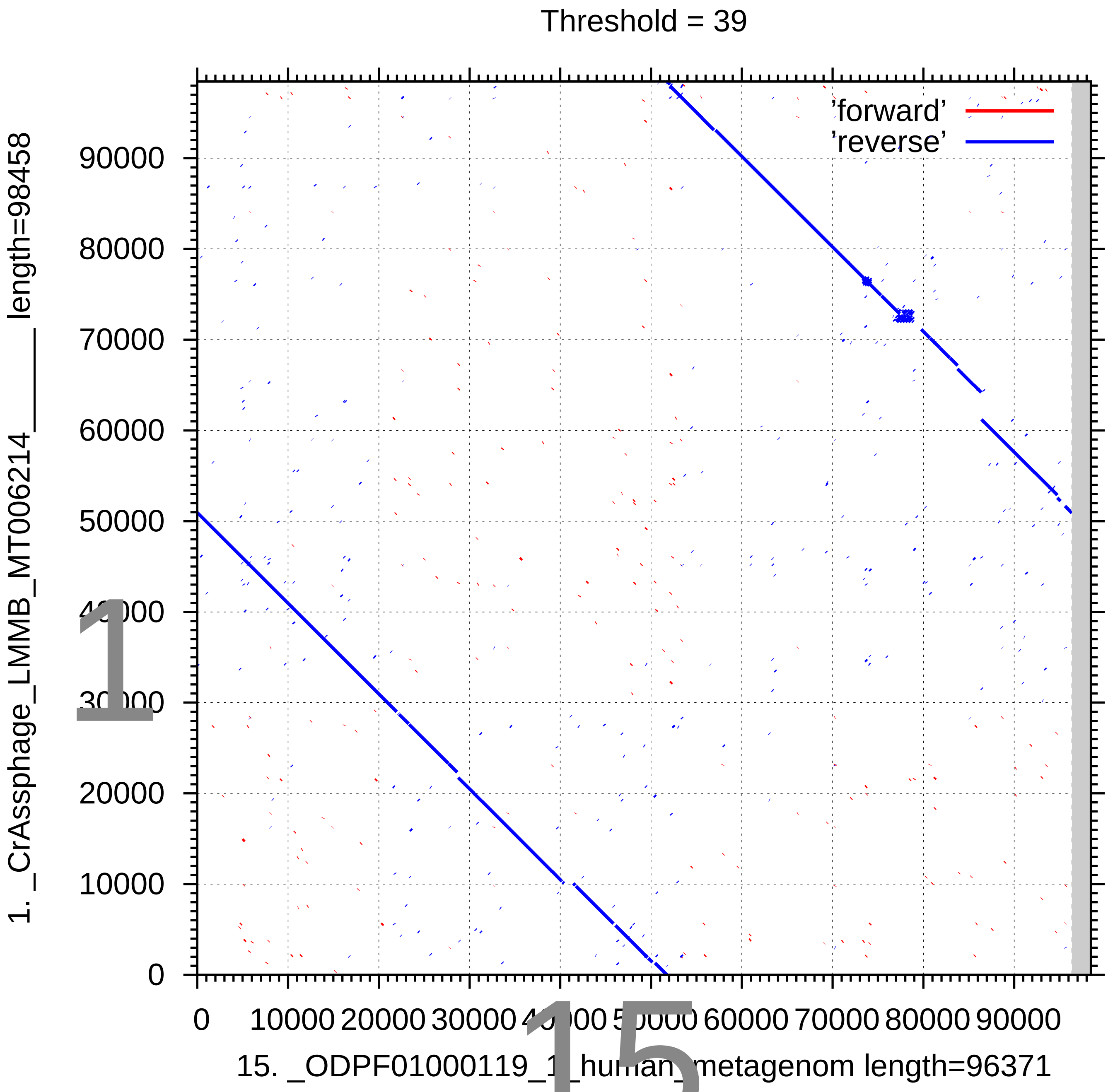

Threshold = 39
'forward'
'reverse'
0
10000
20000
30000
40000
50000
60000
70000
80000
90000
1. _CrAssphage_LMMB_MT006214_______ length=98458
15. _ODPF01000119_1_human_metagenom length=96371

1
15

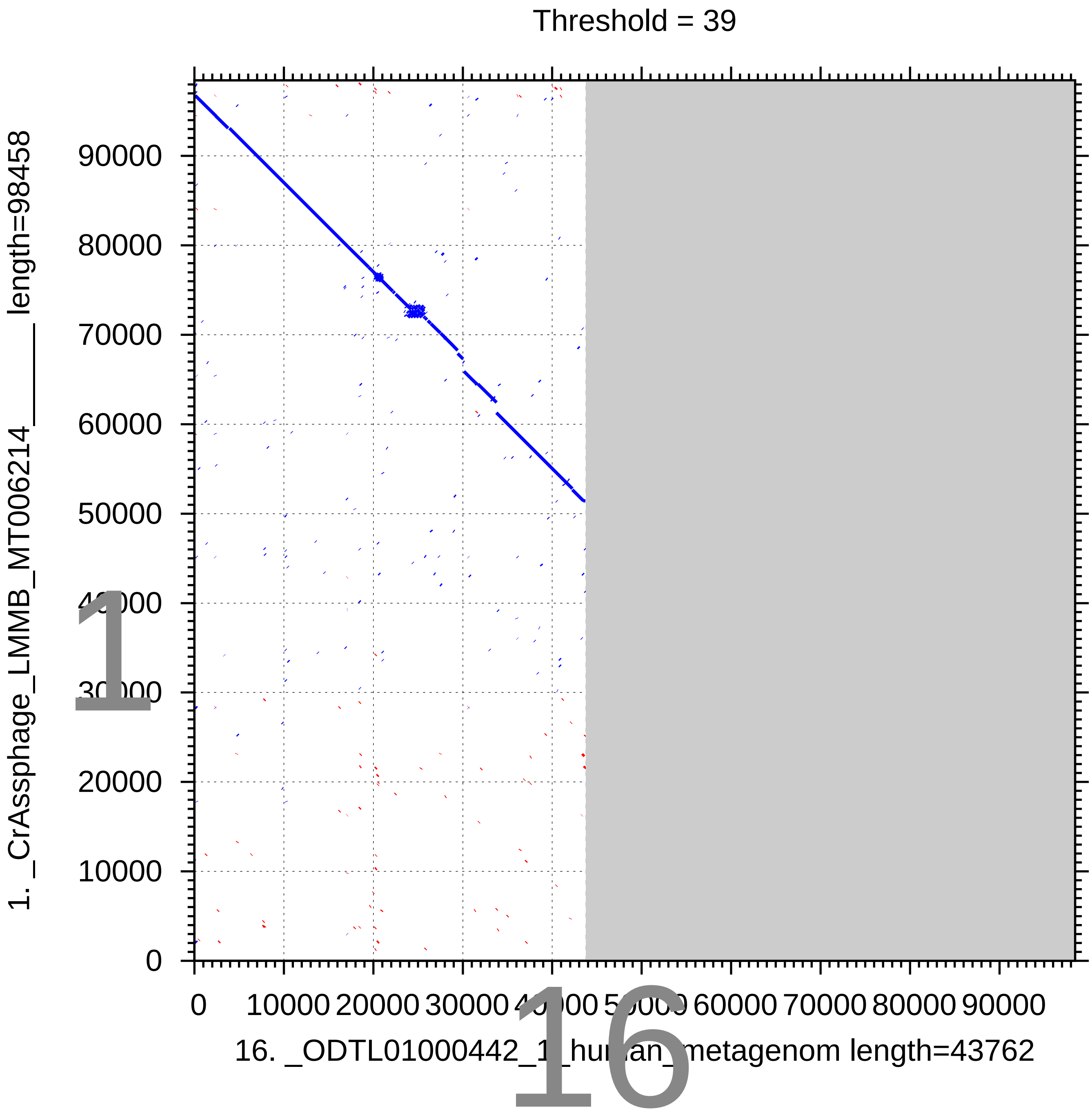
Threshold = 39
1. _CrAssphage_LMMB_MT006214_______ length=98458
16. _ODTL01000442_1_human_metagenom length=43762
0
10000
20000
30000
40000
50000
60000
70000
80000
90000
1
16

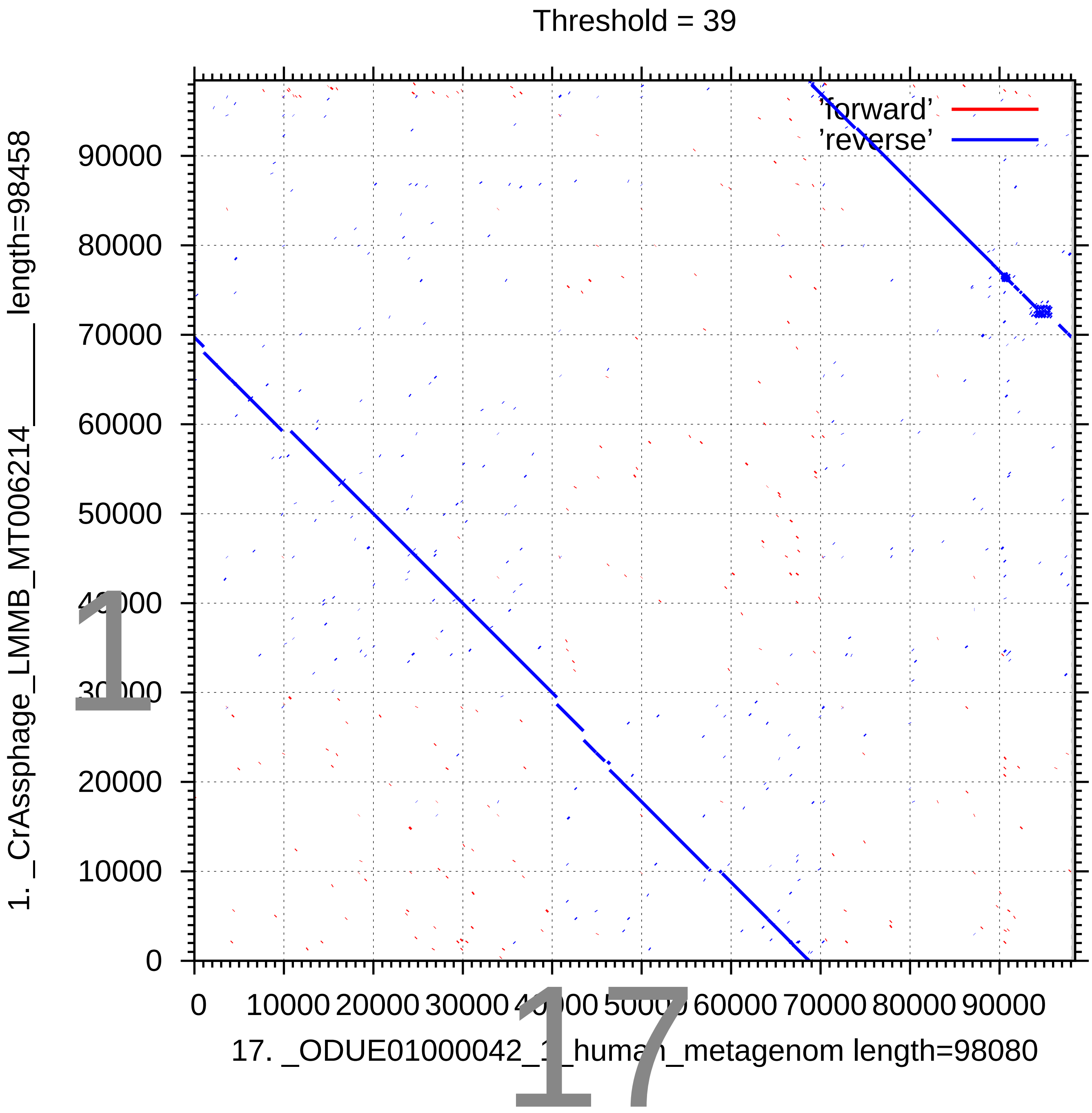

Threshold = 39
'forward'
'reverse'
1. _CrAssphage_LMMB_MT006214_______ length=98458
17. _ODUE01000042_1_human_metagenom length=98080
0
10000
20000
30000
40000
50000
60000
70000
80000
90000
1
17

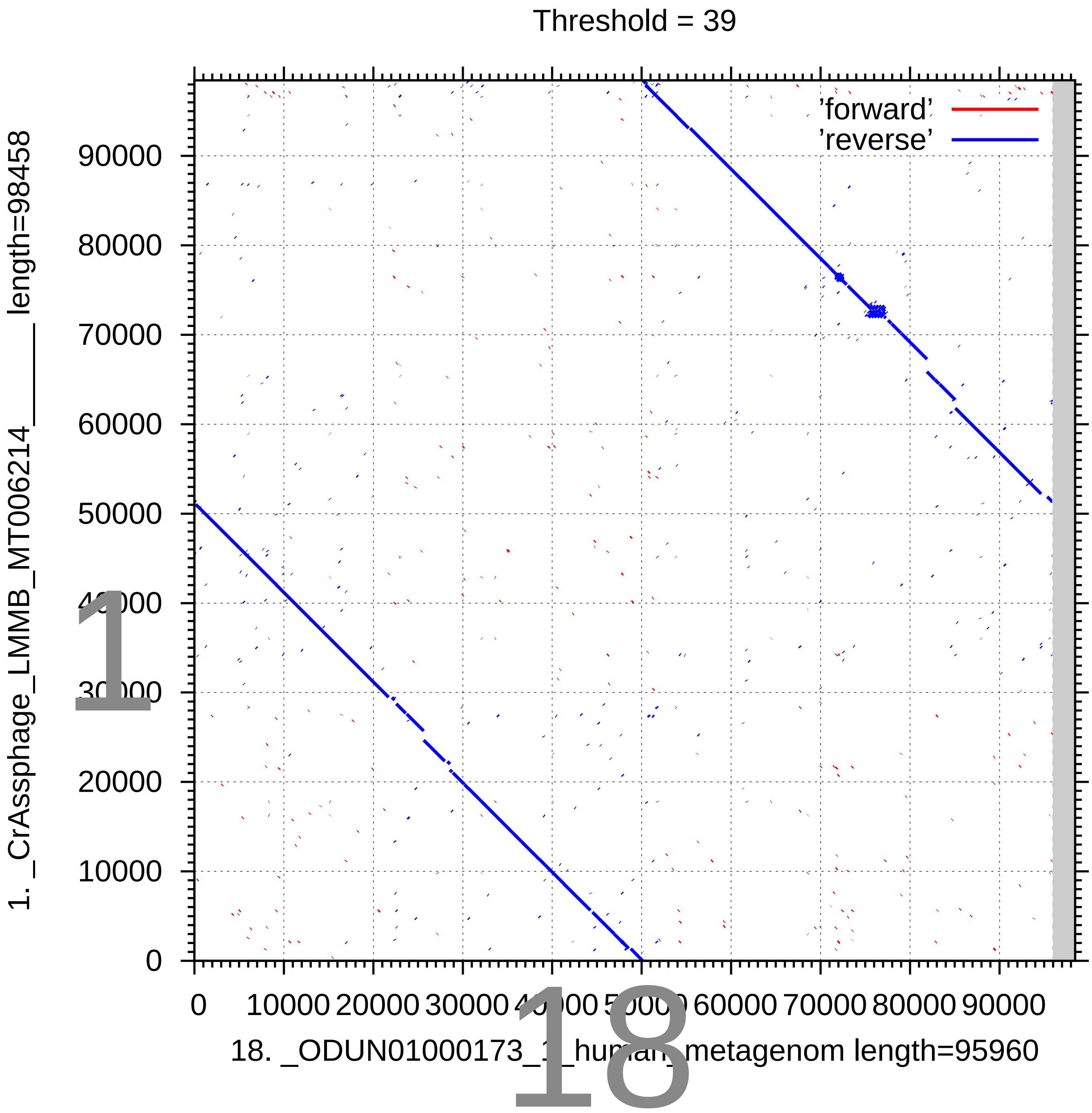
Threshold = 39
'forward'
'reverse'
1. _CrAssphage_LMMB_MT006214_____ length=98458
18. _ODUN01000173_1_human_metagenom length=95960
0
10000
20000
30000
40000
50000
60000
70000
80000
90000
1
18

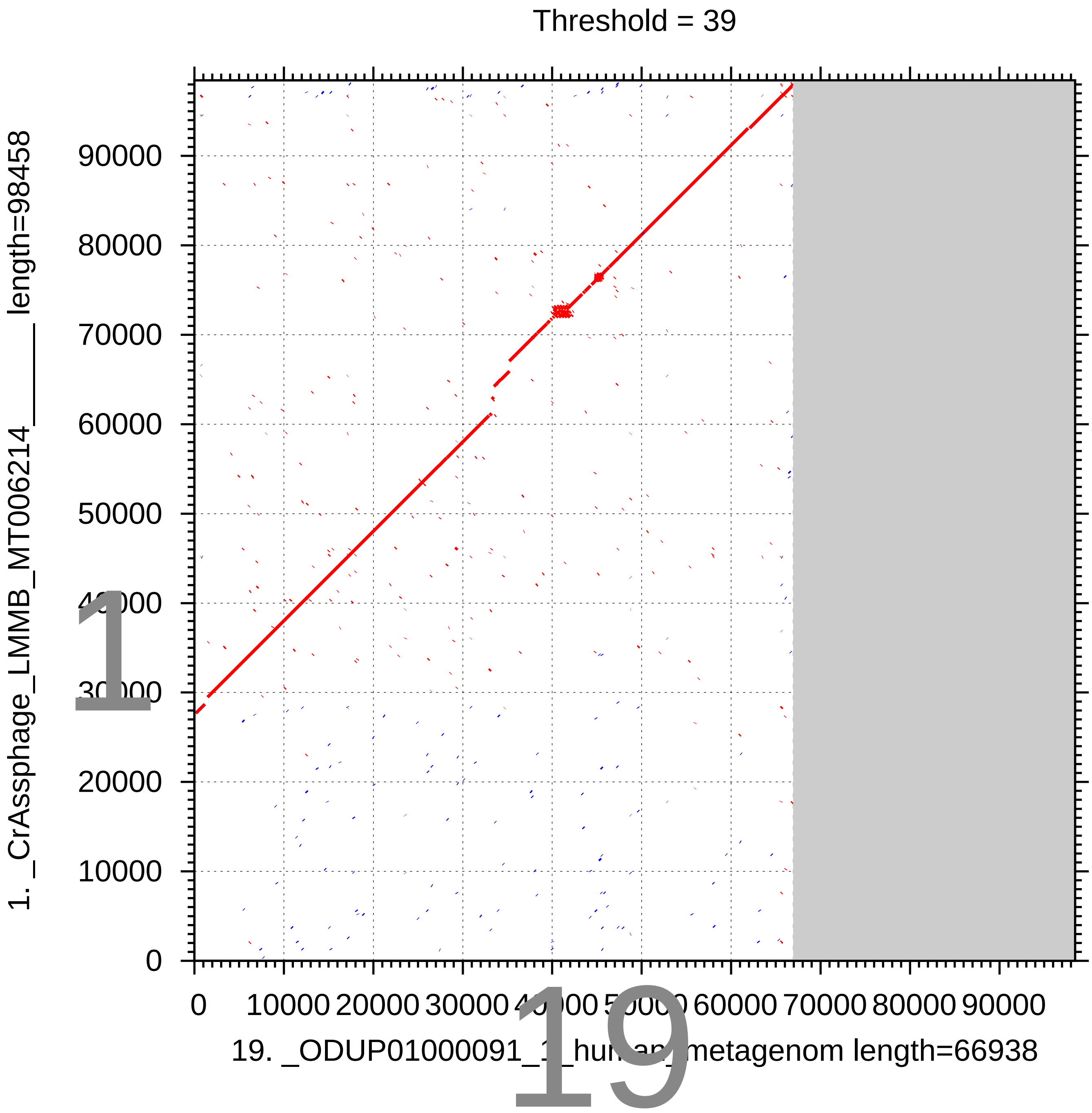
Threshold = 39
1. _CrAssphage_LMMB_MT006214_______ length=98458
0
10000
20000
30000
40000
50000
60000
70000
80000
90000
19. _ODUP01000091_1_human_metagenom length=66938
1
19

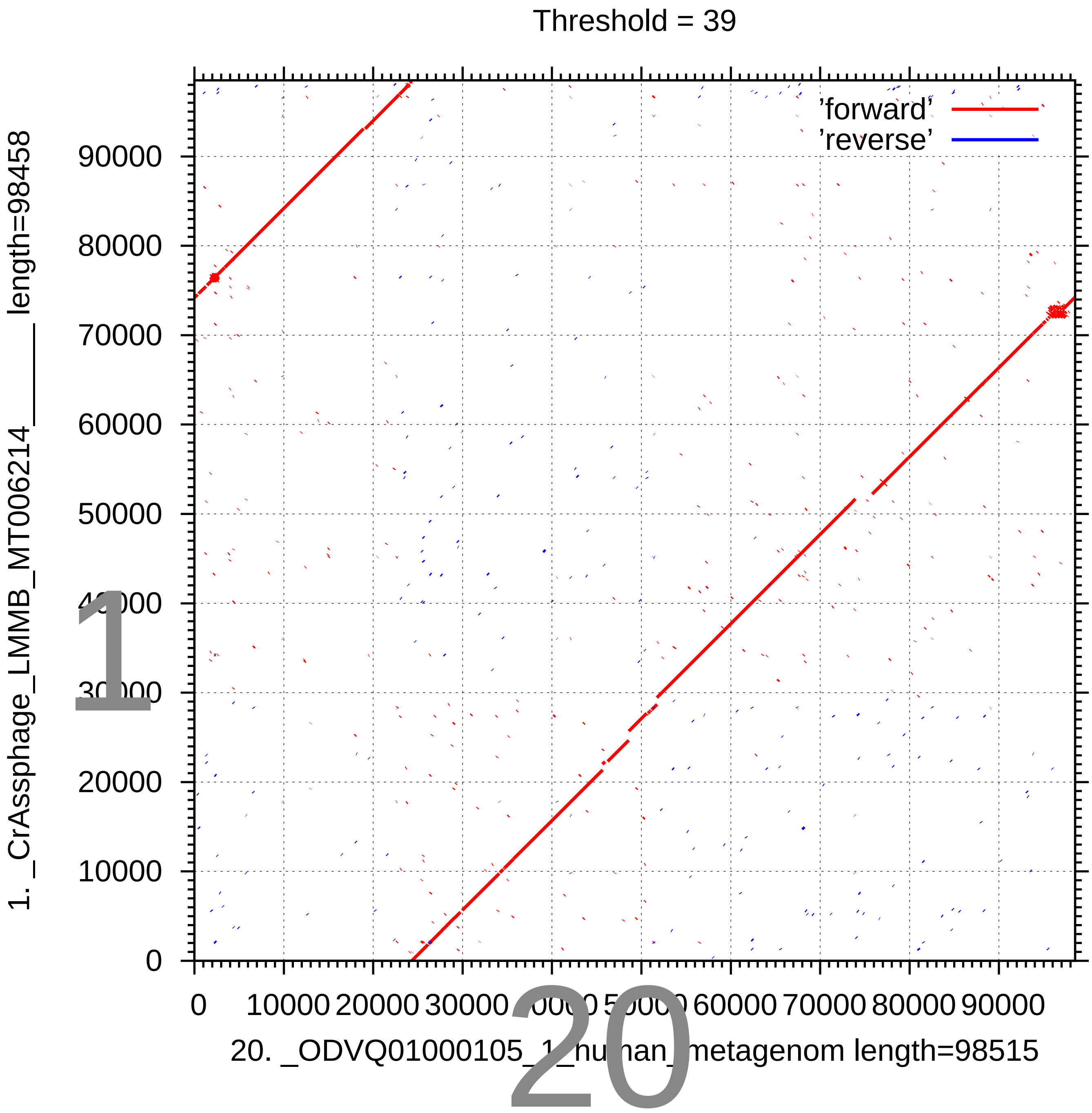

Threshold = 39
'forward'
'reverse'
90000
80000
70000
60000
50000
40000
30000
20000
10000
0
0
10000
20000
30000
40000
50000
60000
70000
80000
90000
1. _CrAssphage_LMMB_MT006214_______ length=98458
20. _ODVQ01000105_1_human_metagenom length=98515
1
20

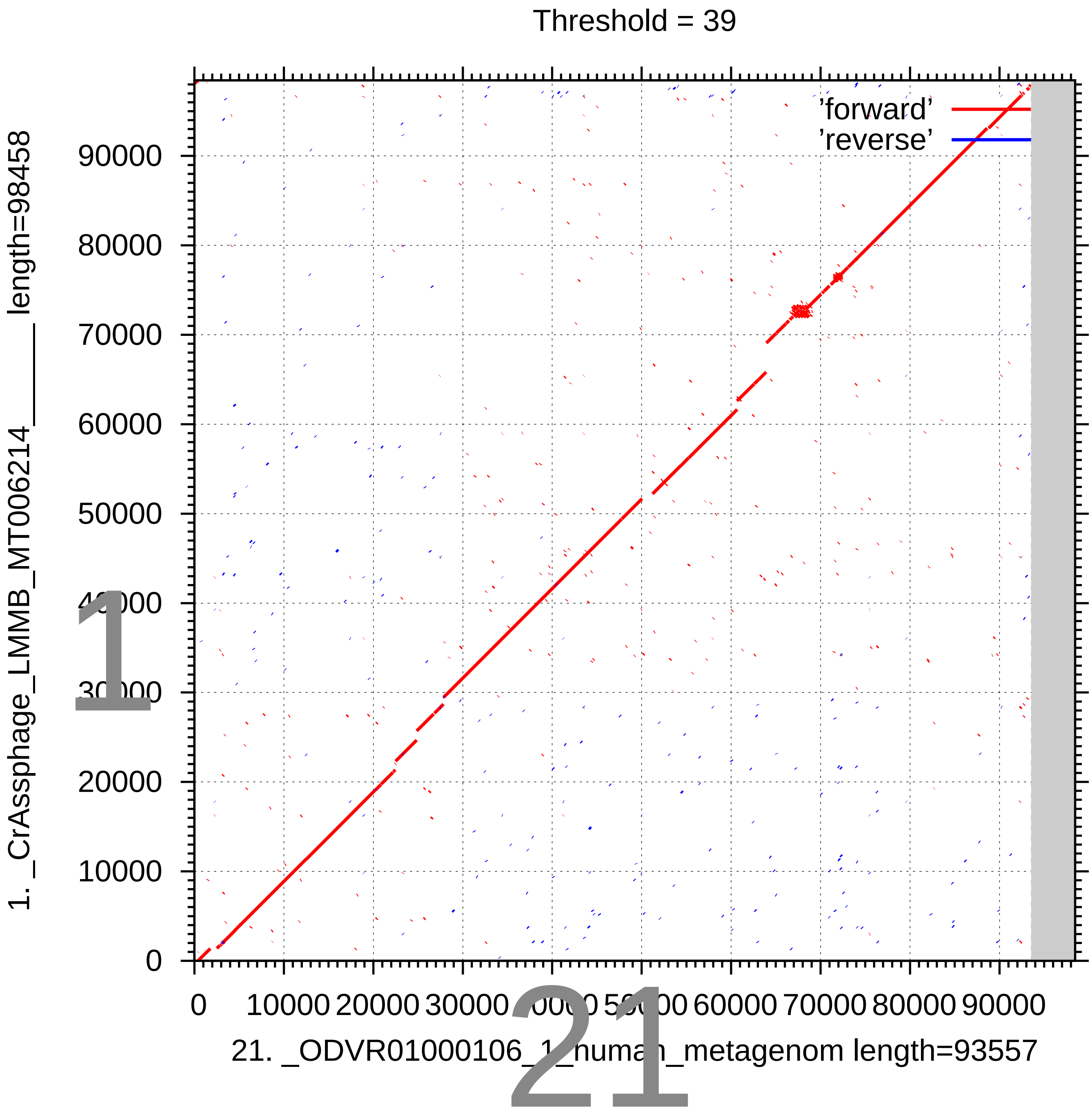

Threshold = 39
'forward'
'reverse'
0
10000
20000
30000
40000
50000
60000
70000
80000
90000
1. _CrAssphage_LMMB_MT006214_______ length=98458
21. _ODVR01000106_1_human_metagenom length=93557
1
21

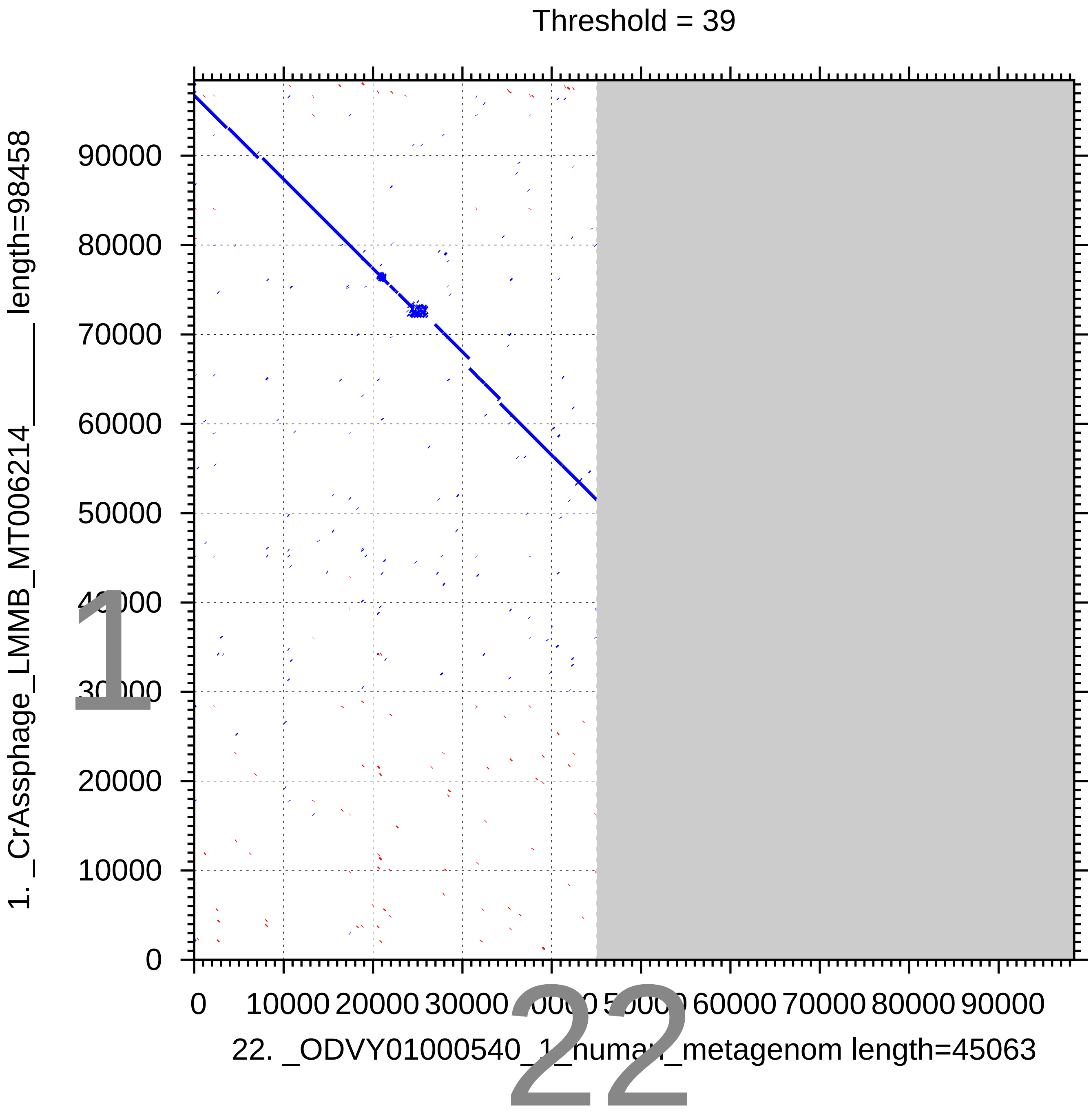
Threshold = 39
1. _CrAssphage_LMMB_MT006214_______length=98458
22. _ODVY01000540_1_human_metagenom length=45063
0
10000
20000
30000
40000
50000
60000
70000
80000
90000
1
22

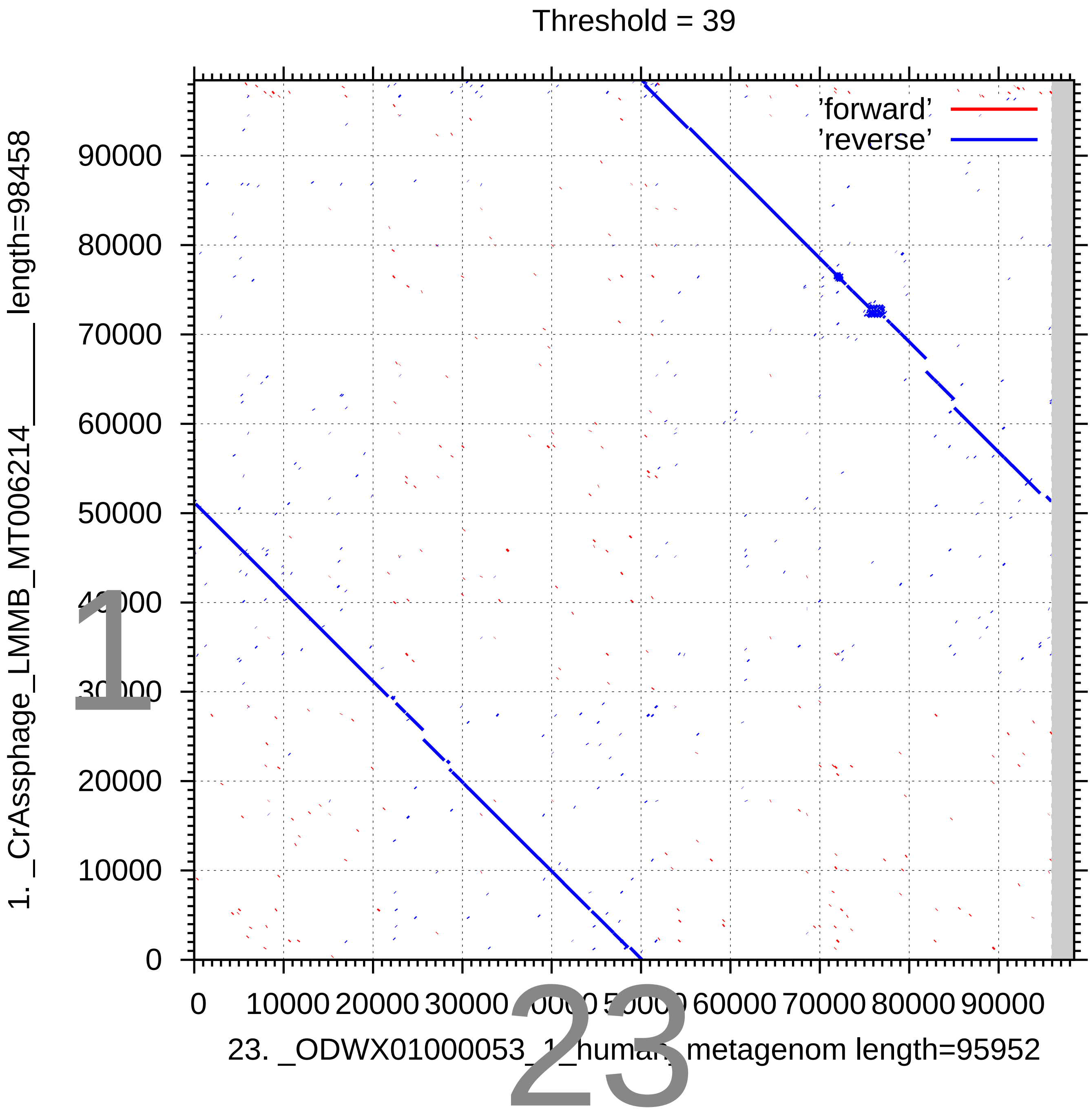
Threshold = 39
'forward'
'reverse'
1. _CrAssphage_LMMB_MT006214 length=98458
23. _ODWX01000053_1_human metagenom length=95952
0
10000
20000
30000
40000
50000
60000
70000
80000
90000
1
23

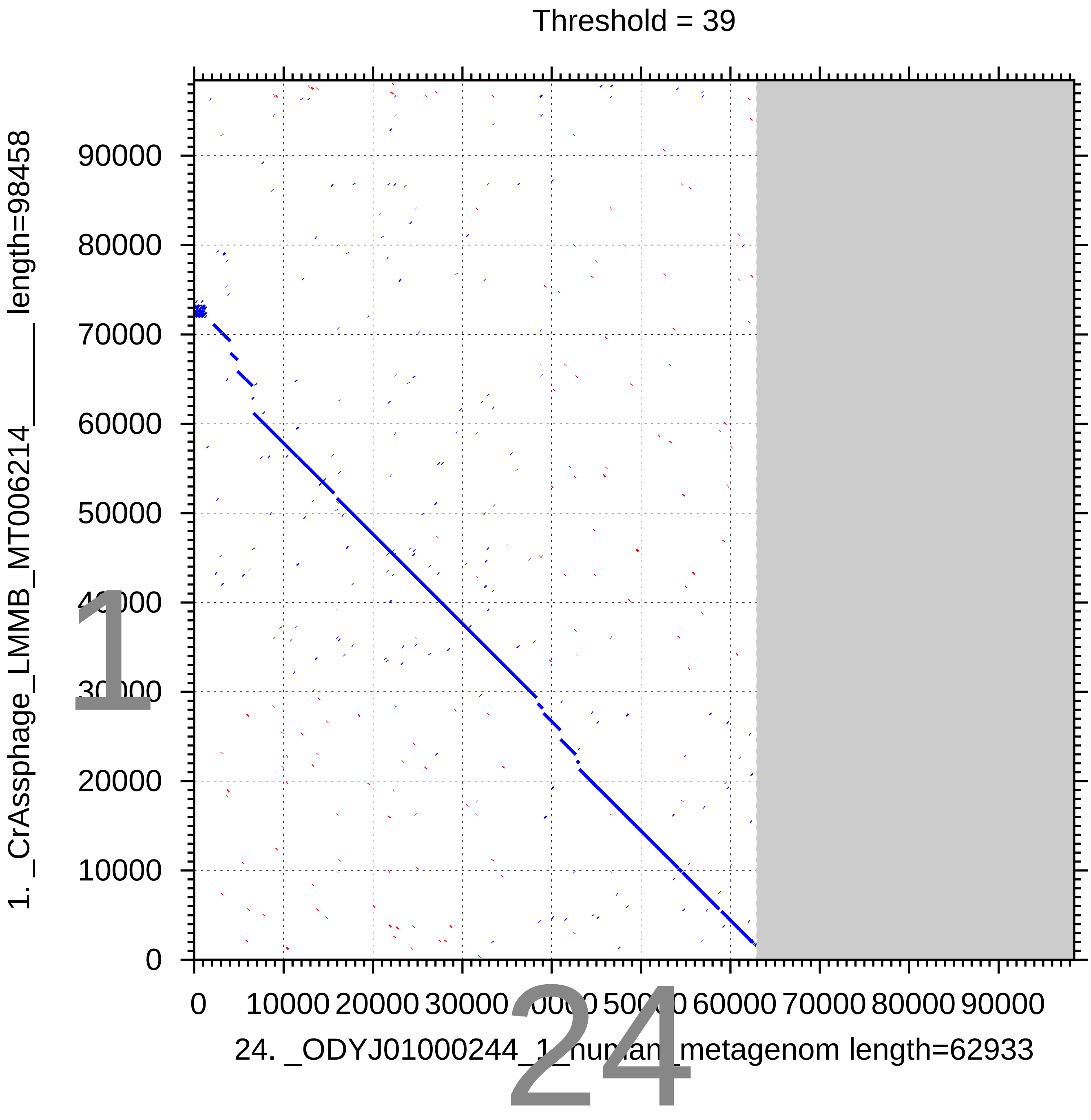

Threshold = 39
1. _CrAssphage_LMMB_MT006214_______ length=98458
0
10000
20000
30000
40000
50000
60000
70000
80000
90000
0
10000
20000
30000
40000
50000
60000
70000
80000
90000
24. _ODYJ01000244_1_human_metagenom length=62933
1
24

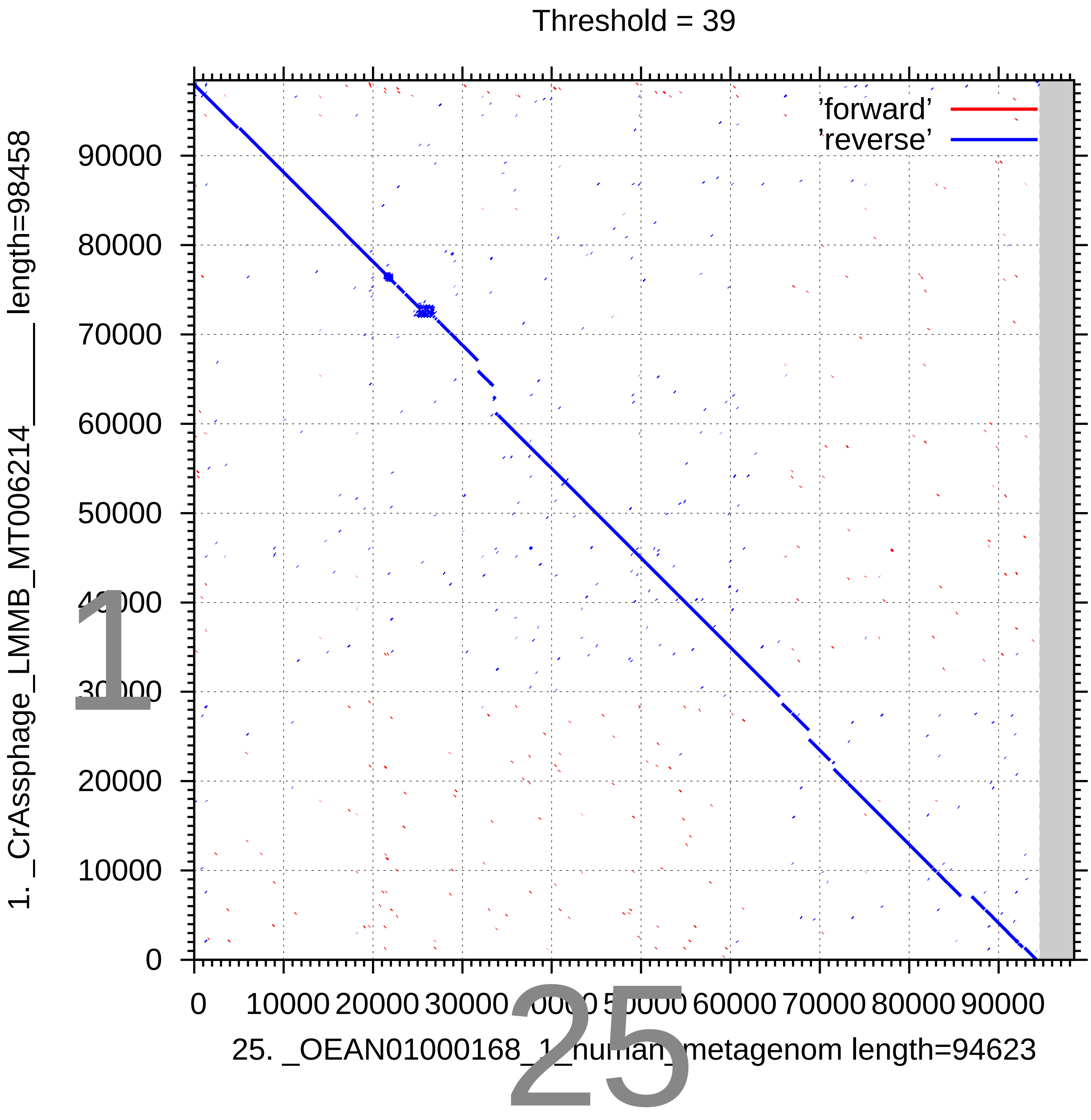
Threshold = 39
'forward'
'reverse'
1. _CrAssphage_LMMB_MT006214 ______ length=98458
0
10000
20000
30000
40000
50000
60000
70000
80000
90000
25. _OEAN01000168_1_human_metagenom length=94623
1
25

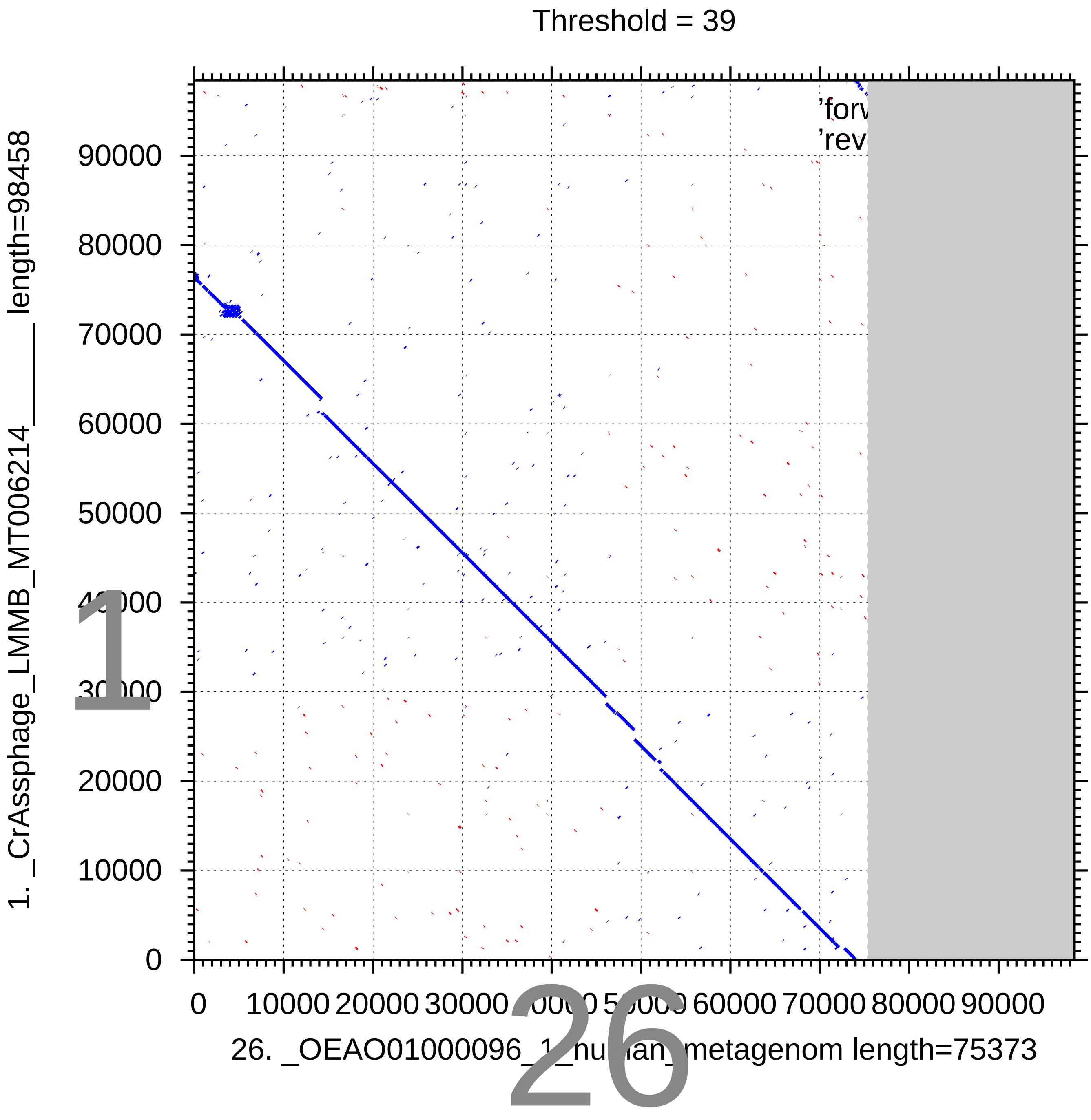

Threshold = 39
'forw
'rev
1. _CrAssphage_LMMB_MT006214________length=98458
26. _OEAO01000096_1_human_metagenom length=75373
0
10000
20000
30000
40000
50000
60000
70000
80000
90000
1
26

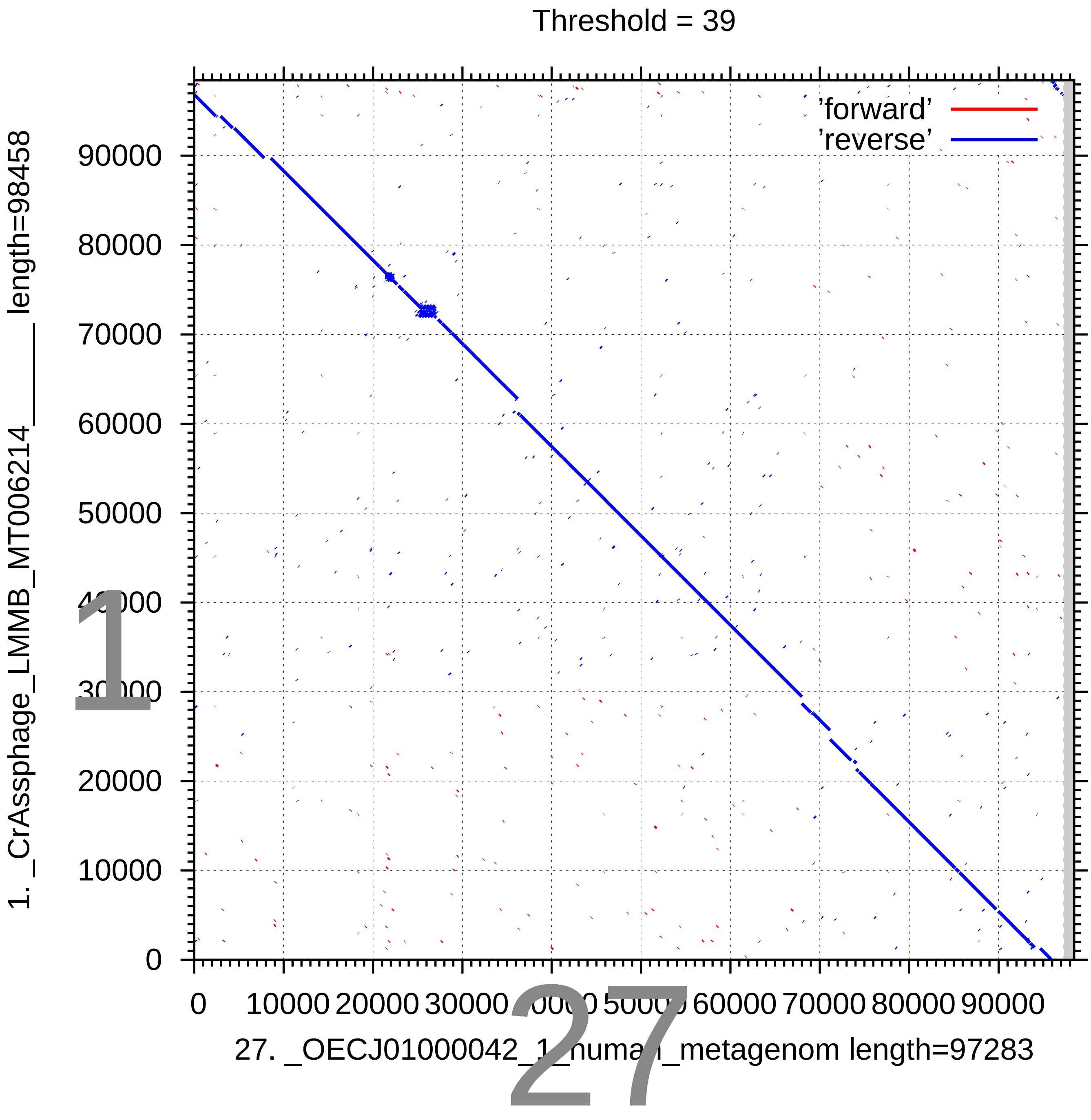

Threshold = 39
'forward'
'reverse'
1. _CrAssphage_LMMB_MT006214_____ length=98458
0
10000
20000
30000
40000
50000
60000
70000
80000
90000
27. _OECJ01000042_1_human_metagenom length=97283
1
27

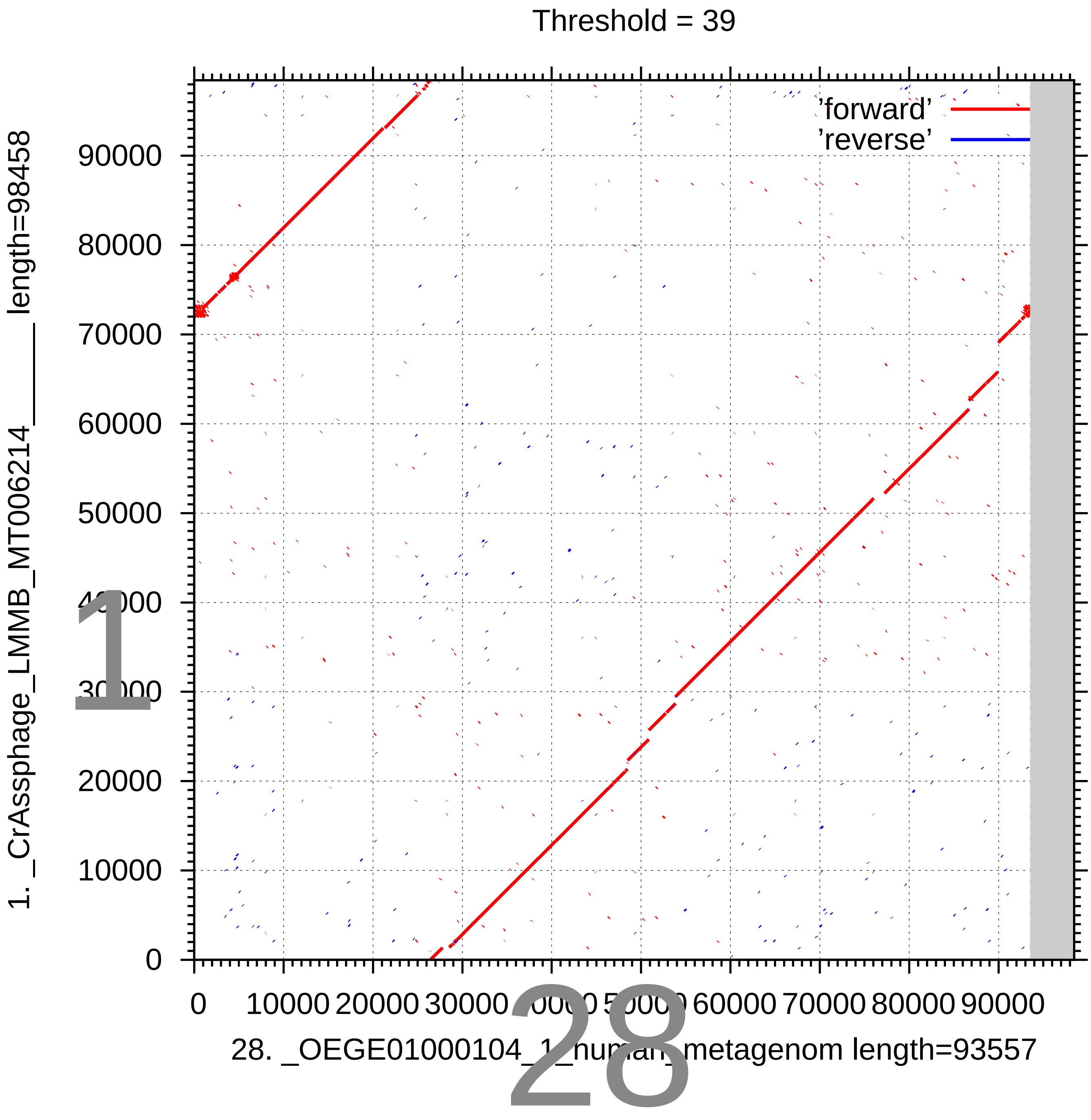

Threshold = 39
'forward'
'reverse'
0
10000
20000
30000
40000
50000
60000
70000
80000
90000
28. _OEGE01000104_1_human_metagenom length=93557
1. _CrAssphage_LMMB_MT006214_______ length=98458
1
28

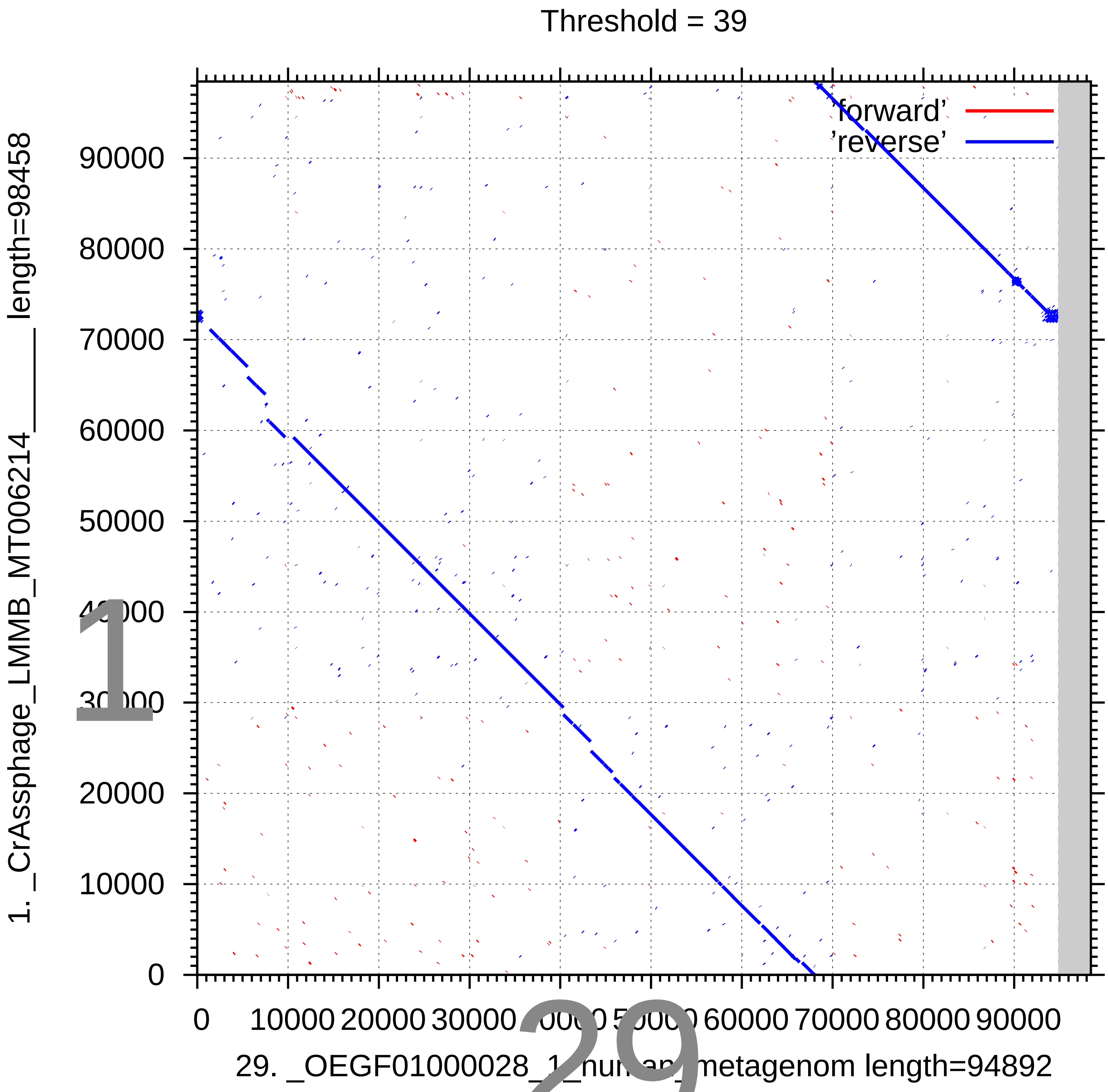

Threshold = 39
'forward'
'reverse'
1. _CrAssphage_LMMB_MT006214_______ length=98458
29. _OEGF01000028_1_human_metagenom length=94892
0
10000
20000
30000
40000
50000
60000
70000
80000
90000

1
29
