# Supplementary material for: First crAss-Like Phage Genome Encoding the Diversity-Generating Retroelement (DGR)
Source: Viruses. 2020 May 22;12(5):573. doi: 10.3390/v12050573 (PMC7290462; doi:10.3390/v12050573)
Supplement: Supplementary file 1 [file viruses-12-00573-s001.zip › Fig. S2.pdf]

```
                          10        20        30        40        50        60        70        80        90        100
                  ....|....|....|....|....|....|....|....|....|....|....|....|....|....|....|....|....|....|....|....|....|
TR LMMB              gtgcttctgcttagcagtaacgctaataacagttctaaagctagctctagtaactttaattctaattgagttcagtcagattctaatgctaatgtcggcttcttt
TR cs_ms_29          .....a...g........................................................................................a........
TR eld314-t0_s_2     .........................................................................................................
TR err843979_ms_2    .......................................................................................a.................
TR err843983_ms_1    .........................................................................................................
TR err844008_ms_2    .....................................................................a..........................a........
TR err844022_ms_1    ............g..g.........................................................................................
TR err844035_ms_4    .........................................................................................................
TR err844045_ms_1    .........................................................................................................
TR err844050_ms_1    .........................................................................................................
TR err844054_ms_2    .............................t...........................................................................
TR err975089_s_1     .........................................................................................................
TR fferm_ms_6        .....a...g........................................................................................a........
TR hvcf_a8_ms_1      .....a...a.......................................................................................t.........
TR hvcf_c9_ms_3      .............................................................................................ta..........
TR hvcf_d10_ms_2     .....................a...................................................................................
TR hvcf_d3_ms_2      .....a...g........................................................................................a........
TR hvcf_d6_ms_5      .........................................................................................................
TR hvcf_e8_ms_1      .............................................................................................ta..........
TR hvcf_f2_ms_2      .........................................................................................................
TR inf008_s_1        .....................a...................................................................................
TR inf058_s_2        .........................................................................................................
TR ODEE01000202.1    .........................................................................................................
TR ODHP01000097.1    .............................t...........................................................................
TR ODIH01000141.1    .........................................................................................................
TR ODTL01000442.1    .........................................................................................................
TR ODUE01000042.1    .............................................................................................a...........
TR ODVQ01000105.1    .............................t...........................................................................
TR ODVR01000106.1    .........................................................................................................
TR OEGE01000104.1    .........................................................................................................
TR srr073438_s_1     .........................................................................................................

                          10        20        30        40        50        60        70        80        90        100
                  ....|....|....|....|....|....|....|....|....|....|....|....|....|....|....|....|....|....|....|....|....|
VR LMMB              atgcttctgcttggcggtcgcgctcattccggttctcgggctggctctggttactttttttctacttgggttcagtcagcttttgatgctactgtcggcttcttt
VR cs_ms_29          g....g...g........g.....t..a.t.......a..............g....aa.....t.......g...g.a..c..c....ca..............
VR eld314-t0_s_2     g.................a.....gc.ca.......at...................g.ca....t.........g...g.g..c.......ta..............
VR err843979_ms_2    ..................a.....ag.ga.......ga.............gg....gc....ga.........g.c.g.a..c.a.....ct..............
VR err843983_ms_1    g..........g......g.....a..a........ga...................ga....gg.........g...g....c.tc....g...............
VR err844008_ms_2    g.......................at.ag.......aa.............ag....ca....ca...ca...g.c.g.a..c.ac....gg..............
VR err844022_ms_1    g.................ta....tc.ga.......tt...................aa....ta.........g...g.g..c.a.....ca..............
VR err844035_ms_4    g......g..........t.....ag.ca.......tc..........a..gg....ga....g..........g.....t..c.ac....ta..............
VR err844045_ms_1    g.................ac..t.g..ag........g..............gg....aa....ca.........g...g.g..c.t....................
VR err844050_ms_1    gc................g.....gg.ca.......ga....................a....t..........g...g.a..c.ac....gg..............
VR err844054_ms_2    g.........g.............g..gat......t...............gg....gc....ga.........g...gta..c.tc....c...............
VR err975089_s_1     g.................ta...........cg.......gc...................ac....ta.........g...g.a..c..c....tt..............
VR fferm_ms_6        g....g.g.g........t.....g..ag.a.....t..............ct.....a..............g...gaa..c..c....tt..............
VR hvcf_a8_ms_1      g................g.........gg..a....ttt.t.......a..ag.....a.....g.....c........a....a......a.............
VR hvcf_c9_ms_3      g.................ta....g..cg.......tc.............c.....ga....ga.........g...g.a..a..........tg..........
VR hvcf_d10_ms_2     g.................ta.......aa.......gc............ggg....aa....cg.........g...g.a..c..g....g...............
VR hvcf_d3_ms_2      g....g...g..a..t..ga....g..g............................g.c....ga.........g...g....c.ag....ta..............
VR hvcf_d6_ms_5      g.................a.....g..aa.......t.a...............c..aa....ga.........g...g.a..c.......ta..............
VR hvcf_e8_ms_1      g.......................g...t.......t..............gg....ca....gg....a....g...g.a..c.cg....ta.tg..........
VR hvcf_f2_ms_2      g......g................at.aa.......ga.............g.....cg....ga.........g...g.a..c..t....ca......g......
VR inf008_s_1        g.................ga.......aa.......g..............gg.....a....gg.........g...gt...c.ac....g...............
VR inf058_s_2        g.......................a...a.......tt..............g.....a....c..........g...g.g..c.......ga..............
VR ODEE01000202.1    g.................g.....ag.at.......t..............c......a.....a.........g...g.a..c..c...................
VR ODHP01000097.1    g.................a.....g..gata.....t..............g......a....ga......a..g...g.a..a.ag....ga..............
VR ODIH01000141.1    g..................t....ag.aa.......g....................a.....ca.........g..tgta..c..g....ca..............
VR ODTL01000442.1    g.......................gc.gg.......ga.............c......a.....g.....g..g.....t..c..c....caa.............
VR ODUE01000042.1    g.................g.....g..ga.......a..............gg.....a....ta.........g...g.a..c.ac.....a..............
VR ODVQ01000105.1    g.................a.....tt.g.t......tc...................ca....cg.........g...g.a..c.tc....ta..............
VR ODVR01000106.1    g......g.......a........g..ga.t......t...................ca....tg.........g...g.a..c.c.g...g...............
VR srr073438_s_1     g.......................g..ga.......gc.............g.....ca....g..........g...gaa..c..c....ta..............
```
